# Supplementary figures and images for: Prevalence of Noncommunicable Disease (NCDs) risk factors in Tamil Nadu: Tamil Nadu STEPS Survey (TN STEPS), 2020
Source: PLoS One. 2024 May 8;19(5):e0298340. doi: 10.1371/journal.pone.0298340 (PMC11078398; doi:10.1371/journal.pone.0298340)

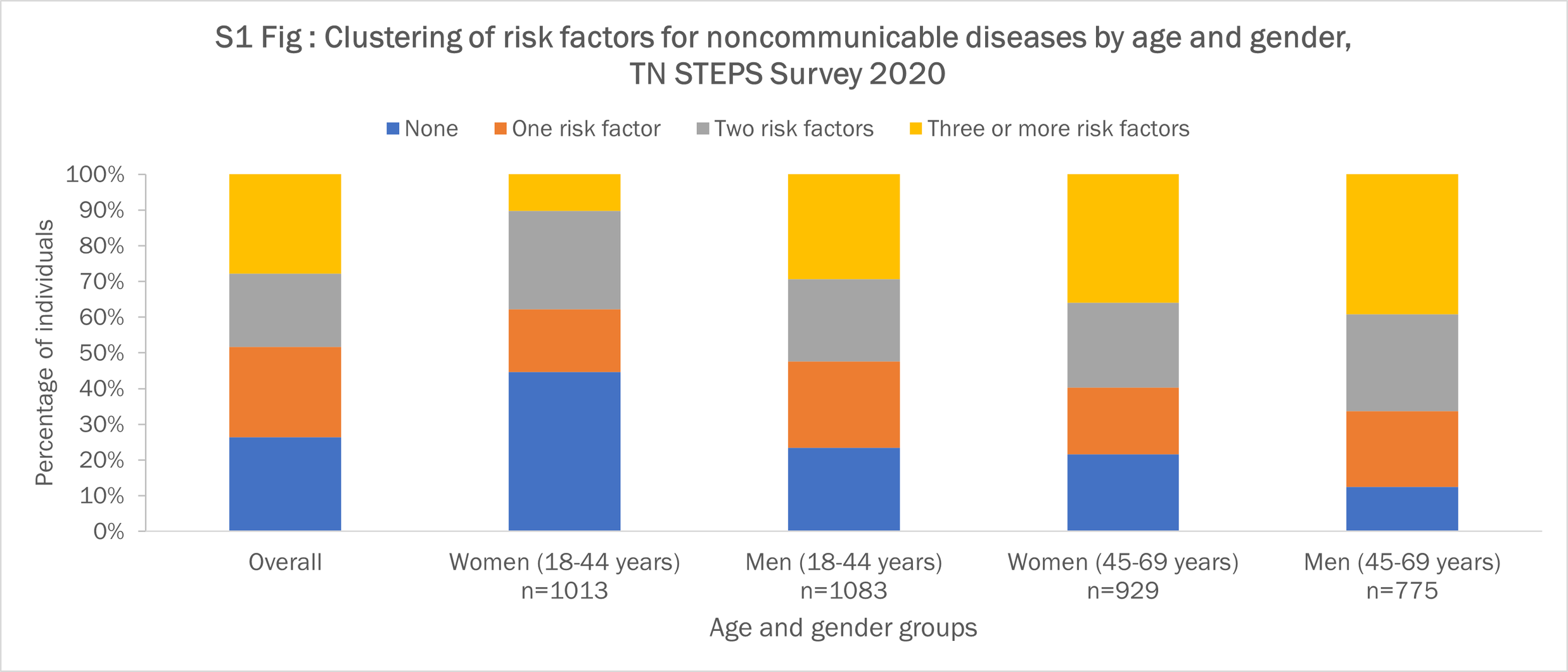

Supplement: S1 Fig — (TIF) [file pone.0298340.s001.tif]
