# Supplementary material for: Prevalence of Noncommunicable Disease (NCDs) risk factors in Tamil Nadu: Tamil Nadu STEPS Survey (TN STEPS), 2020
Source: PLoS One. 2024 May 8;19(5):e0298340. doi: 10.1371/journal.pone.0298340 (PMC11078398; doi:10.1371/journal.pone.0298340)
Supplement: S1 Table — (DOCX) [file pone.0298340.s002.docx]

**S1 Table: Multicollinearity test using Variation Inflation Factor (VIF) for Hypertension and Diabetes.**

|  | Hypertension | Diabetes |
| --- | --- | --- |
| Variable | VIF | VIF |
| Age | 1.08 | 1.12 |
| Gender | 1.47 | 1.48 |
| Currently smoking | 1.29 | 1.28 |
| Current alcohol use | 1.45 | 1.46 |
| Uses Added salt (always ) | 1 | 1 |
| Physical inactivity | 1.04 | 1.03 |
| Central Obesity | 1.88 | 1.88 |
| Body Mass Index |  |  |
| 25-29.9 | 1.62 | 1.62 |
| >=30 | 1.52 | 1.53 |
| Diabetes | 1.11 | - |
| Hypertension | - | 1.19 |
