# Supplementary material for: Prevalence of Noncommunicable Disease (NCDs) risk factors in Tamil Nadu: Tamil Nadu STEPS Survey (TN STEPS), 2020
Source: PLoS One. 2024 May 8;19(5):e0298340. doi: 10.1371/journal.pone.0298340 (PMC11078398; doi:10.1371/journal.pone.0298340)
Supplement: S2 Table — (DOCX) [file pone.0298340.s003.docx]

**S2 Table: Predictors of NCD risk factors* among study participants of the Tamil Nadu STEPS Survey, 2020.**

| **Characteristics** | | **Smoking** | **Alcohol** | **Physical Activity** | **Abdominal Obesity** |
| --- | --- | --- | --- | --- | --- |
|  |  | **aPR †** | **aPR †** | **aPR †** | **aPR †** |
| Age | 18-44 | reference | reference | reference | reference |
|  | 45-69 | 0.9 (0.8-1.1) | 0.8 (0.7-0.9) | 0.97 (0.96-0.99) | 1.3 (1.2-1.4) |
| Gender | Female | - | - | 1.05 (1.04-1.07) | 1.4 (1.3-1.6) |
|  | Male | - | - | reference | reference |
| Marital status | Never married | reference | reference | reference | reference |
|  | Currently married | 1.6 (1.2-2.2) | 1.1 (0.9-1.3) | 1.03 (1.003-1.05) | 1.7 (1.3-2.1) |
|  | Divorced/separated | 1.6 (1-2.7) | 1 (0.7-1.5) | 1.05 (1.02-1.08) | 1.6 (1.3-2.1) |
| Occupation | Professional/ Agri landowner/sales or marketing/officers | reference | reference | 1.0001 (0.98-1.02) | 0.9 (0.8-1.1) |
|  | Business | 0.9 (0.7-1.2) | 1 (0.8-1.2) | 0.98 (0.95-1.01) | 1.1 (0.9-1.2) |
|  | Labourer | 1.1 (0.9-1.4) | 1.2 (1-1.4) | 1.03 (1.01-1.04) | 0.7 (0.7-0.8) |
|  | Homemaker | - | - | reference | reference |
|  | Others | 1.1 (0.8-1.5) | 0.9 (0.7-1.1) | 0.97 (0.94-0.99) | 0.8 (0.7-0.9) |
| Place of residence | Urban | 1.1 (1-1.3) | 0.9 (0.8-1.1) | 0.99 (0.97-1.01) | 1.2 (1.1-1.4) |
|  | Rural | reference | reference | reference | reference |
| Education | Illiterate | 2.8 (2-3.9) | 2.1 (1.6-2.7) | reference | reference |
|  | Primary | 1.9 (1.4-2.5) | 1.7 (1.4-2.1) | 1.001 (0.98-1.02) | 1.2 (1.1-1.3) |
|  | Secondary | 1.7 (1.2-2.4) | 1.4 (1-1.8) | 0.98 (0.96-1.01) | 1.3 (1.1-1.5) |
|  | Graduation & above | reference | reference | 0.98 (0.96-1) | 1.3 (1.1-1.5) |
| * NCD risk factors included predictors of smoking & alcohol among men and predictors of physical activity & abdominal obesity among all individuals. | | | | | |
| † Adjusted for all factors significant in univariable analysis and key confounders based on literature | | | | | |
