# Supplementary material for: Prevalence of Noncommunicable Disease (NCDs) risk factors in Tamil Nadu: Tamil Nadu STEPS Survey (TN STEPS), 2020
Source: PLoS One. 2024 May 8;19(5):e0298340. doi: 10.1371/journal.pone.0298340 (PMC11078398; doi:10.1371/journal.pone.0298340)
Supplement: S3 Table — (DOCX) [file pone.0298340.s004.docx]

### S3 Table**: Responders and Non-responders of the TN STEPS Survey, 2020 by age and gender**

| Characteristics |  | Non-respondents | | Respondent | |
| --- | --- | --- | --- | --- | --- |
|  |  | n | % | n | % |
| Gender | Male | 234 | 72 | 1,858 | 49 |
|  | Female | 91 | 28 | 1,942 | 51 |
| Age | 18-44 | 215 | 66 | 2,096 | 55 |
|  | 45-69 | 110 | 34 | 1,704 | 45 |
