# Supplementary material for: Prevalence of Noncommunicable Disease (NCDs) risk factors in Tamil Nadu: Tamil Nadu STEPS Survey (TN STEPS), 2020
Source: PLoS One. 2024 May 8;19(5):e0298340. doi: 10.1371/journal.pone.0298340 (PMC11078398; doi:10.1371/journal.pone.0298340)
Supplement: S1 File — (DOC) [file pone.0298340.s005.doc]

**S1 File: STEPS survey for NCDs in Tamil Nadu, 2020 Questionnaire**

Survey Questionnaire

ID

Individual

|  |  |  |  |  |
| --- | --- | --- | --- | --- |

**STEPS survey for NCDs in Tamil Nadu, 2020**

**STEPS - தமிழ் நாட்டில் தொற்றா நோய்களுக்கான கணக்கெடுப்பு, 2020**

# Question by Question Guide

# கேள்வி வாரியான வழிகாட்டி

**Overview**

**Introduction:** The Question-by-Question Guide presents the questionnaire with a brief explanation for each of the questions.

**Purpose:** The purpose of the Question-by-Question Guide is to provide background information to the interviewers and supervisors as to what is intended by each question. Interviewers can use this information when participants request clarification about specific questions or they do not know the answer. Interviewers and supervisors should refrain from offering their own interpretations.

**Guide to the columns**

The table below is a brief guide to each of the columns in the Q-by-Q Guide

| **Column** | **Description** | **Site Tailoring** |
| --- | --- | --- |
| Question | Each question is to be read to the participants | Select sections to use |
| Response | This column lists the available response options which the interviewer will be filling in the text boxes. The skip instructions are shown on the right-hand side of the responses and should be carefully followed during interviews. | Change skip question  identifiers where necessary |
| Code | The column is designed to match data from the instrument into the data entry tool, data analysis syntax, data book, and fact sheet. | This should never be changed  or removed. The code is used  as a general identifier for the  data entry and analysis |

**General Instruction to interviewer**

- *Understand what you are going to ask and what are you going to re*cord. All instructions are in italics (these are for you to understand), instructions to be read out to participants are in grey boxes

| Identification to be filled from household form after selecting individual | | | | | | | |
| --- | --- | --- | --- | --- | --- | --- | --- |
| PSU Number*Record PSU code from the list provided* |  |  |  |  | | | |
| Household Number*Enter household number from the list* | |  |  |  | | | |
| Name of the selected adult respondent*Write the name of the person chosen for interview* | | | | ……………………………….. | | | |
| Written Informed Consent obtained*Read out the consent / give the consent form to the participant to read it themselves and if the participant gives consent, obtain signature and record as consent given, else mark as consent not given* | | | | | Yes/ …1No/ …2 |  | *If No, End* |
|  | | |

- *Read each question* ***verbatim****. In case the respondent does not understand you can explain in own language carefully without changing the meaning of the question*
- *Answer codes are provided for each section. However some codes are universal e.g. do not know or do not remember =7or, 77or 777; Refuses information or, no response =8 or, 88 or, 888; Not applicable=9 or, 99or, 999*
- *Skip patterns are provided at relevant points, please follow them*
- *Note the number in the respective boxes given. Each box is for one digit. For single digit response, fill the second/right box in each cell and zero in the left box.*

*Enter the date of visit as directed by the background inside the box. Pick the relevant code from the result column*

| **Visit** | **Date** | **Result** Codes:  Completed = 1, Not at Home = 2, Refused =3, Partly completed =4, Other (Specify) =5 | | | | | |
| --- | --- | --- | --- | --- | --- | --- | --- |
|  |  | **Section 1** | | | **Section 2** | | |
| *First* | | D | D | M | M | Y | Y | | --- | --- | --- | --- | --- | --- | |  |  |  |  |  |  |
| *Second* | | D | D | M | M | Y | Y | | --- | --- | --- | --- | --- | --- | |  |  |  |  |  |  |
| *Third* | | D | D | M | M | Y | Y | | --- | --- | --- | --- | --- | --- | |  |  |  |  |  |  |
| Total Number of Visits (*to be filled at the completion of form, else at the end of the third visit*) Fill this after you are sure you don’t need to visit the house again for filling this form that is either the form has been completed or three visits are over. | | | | |  |  |  |

| **Blood Pressure and Pulse Rate இரத்த அழுத்தம் மற்றும் நாடி துடிப்பு** | | | |
| --- | --- | --- | --- |
| - *For Blood Pressure (BP) measurement the respondent must be comfortable and if the respondent had been exerting then he/she must rest for at least 5 minutes.*   இரத்த அழுத்த அளவீடுகளுக்கு, பேட்டி அளிப்பவர் சௌகரியமாக இருக்க வேண்டும் மற்றும் பேட்டியளிப்பவர் ஏதேனும் கடின வேலை செய்து கொண்டிருந்தால் அவர் குறைந்தபட்சம் 5 நிமிடங்களுக்கு ஓய்வெடுக்க வேண்டும்.   - *There must be at least 5 minutes gap between two consecutive readings.*   *இரண்டு தொடர்ச்சியான அளவீடுகள் இடையே குறைந்தபட்சம் 5 நிமிடங்கள் இடைவெளி இருக்க* வேண்டும்.  *If the respondent refuses any of the measurement write 88 or 888*  *பேட்டியளிப்பவர் ஏதாவது அளவீடுகளைச் செய்வதற்கு மறுத்தால் 88 அல்லது 888 என எழுதவும்* | | | |
|  | **Blood Pressure and Heart Rate 1**  **இரத்த அழுத்த** அளவீடு 1 | **Response**  **பதில்** | |
| M1a | Systolic blood pressure (mmHg)  சிஸ்டோலிக் இரத்த அழுத்தம்  பங்கேற்பாளர் 15 நிமிடங்கள் ஓய்வெடுத்த பின் முதல் அளவீட்டைப் பதிவு செய்யவும். | Systolic (mmHg)  சிஸ்டோலிக் | |  |  |  | | --- | --- | --- | |
| M1b | Diastolic blood pressure (mmHg)  டையாஸ்டோலிக் இரத்த அழுத்தம் | Diastolic (mmHg)  டையாஸ்டோலிக் | |  |  |  | | --- | --- | --- | |
| M1c | Pulse Rate Reading  இதயத்துடிப்புஅளவீடு  Record the reading  அளவீட்டைப் பதிவு செய்யவும் | Beats/min  நாடிதுடிப்புகள் / நிமிடத்திற்கு | |  |  |  | | --- | --- | --- | |

| **Section 1.A Demographic Information பொதுவான குடும்ப தகவல்கள்** | | | | | |
| --- | --- | --- | --- | --- | --- |
| Questions  கேள்விகள் | | Response  பதில் | | | Skip  விட வேண்டியது |
| C1 | Sex of the respondent;  பேட்டியளிப்பவரின் பாலினம்  Write the sex of the individual as observed  கவனிக்கப்பட்டபடி தனிநபரின் பாலினத்தை எழுதவும் | Male…1  ஆண்……….1  Female…2  பெண்…...2 | |  | | --- | | |  |
| C2 | Do you know your date of birth?  உங்கள் பிறந்த தேதி உங்களக்கு தெரியுமா?  Record the date of birth of the person. You cannot enter a partial date of birth. Select "Don't know" in all fields if complete date of birth is not known. | | D | D | M | M | Y | Y | Y | Y | | --- | --- | --- | --- | --- | --- | --- | --- | | | | *If date provided go to C4*  *தேதி அளிக்கப்பட்டிருந்தால், C4க்கு செல்லவும்* |
| C3 | What is your age?  உங்கள் வயது என்ன?  Record the age of the person. If unknown help him/her estimate the age by asking further questions on well known major events or can ask the parents or guardian.  அந்த நபரின் வயதைப் பதிவு செய்யவும். தெரியவில்லை என்றால், நன்கு அறியப்பட்ட முக்கிய நிகழ்வுகள் மீதான மேலும் கேள்விகளை கேட்பதன் மூலமாக அவருடைய வயதை மதிப்பீடு செய்வதற்கு அவருக்கு உதவவும், அல்லது பெற்றோர் அல்லது காப்பாளரிடம் கேட்கலாம். | Age in completed years  பூர்த்தியடைந்த வருடங்களில் வயது | | |  |  | | --- | --- | |  |
| C4 | What is your current marital status?  உங்களின் தற்போதைய திருமணநிலை என்ன?  Select the appropriate response.  பொருத்தமான பதிலைத் தேர்ந்தெடுக்கவும் | Never married ……… 1திருமணமாகாதவர்Living in /co habiting….2சேர்ந்து வாழ்பவர்Currently Married ……….3திருமணமானவர் Separated....4  பிரிந்து வாழ்பவர்  Divorced….5  விவாகரத்தானவர்  Widowed…6  துணையை இழந்தவர்  No response /Do not want to answer…… 8  பதில் இல்லை | | |  | | --- | |  |
| C5 | Have you ever been imparted any education?  எப்பொழுதாவது கல்வி கற்றுள்ளீர்களா? Select the appropriate response.  பொருத்தமான பதிலைத் தேர்ந்தெடுக்கவும் | No……இல்லை……….1Yes in a formal school……ஆம் முறையான பள்ளியில்...2Yes in a madrassa/ Gurukul etc……ஆம் மதராசா/குருகுலம்….3Yes home schooledவீட்டுக் கல்வி …4 | | |  | | --- | | *If No go to C9*  *இல்லை என்றால் C9 செல்லவும* |
| C6 | If yes, what is the highest level of education you completed?  ஆம் என்றால். நீங்கள் அதிகபட்சமாக எதுவரைப் படித்திருக்கிறீர்கள்?  Choose the years of education even if it was not gained in a formal school .e.g. a person who completed 6 years of Madrassa education or 6 years of education in a school or 6 years of education at home all will be coded as “02”  கல்வி வருடங்களை தேர்ந்தெடுக்கவும். ஒரு நபர் மதராசாவில் 6 வருடம் அல்லது பள்ளியில் 6 வருடம் அல்லது வீட்டுக்கல்வியில் 6 வருடம் முடித்திருந்தால் அனைத்தும் 02 ல் குறிக்கவேண்டும்; | Less than Class 6…  6-ம் வகுப்பிற்கும் குறைவானது …. 1  Class 6 or 7 or 8 completed …  வகுப்பு 6 அல்லது 7 அல்லது 8 முடித்திருக்கிறார்....2  Class 9 or 10 completed…  வகுப்பு 9 அல்லது 10 முடித்திருக்கிறார்;...3  Class 11-12 completed...  வகுப்பு 11-12 முடித்திருக்கிறார் …4  Graduation or diploma completed…  பட்டப்படிப்பு அல்லது பட்டயப் படிப்பு முடித்திருக்கிறார் …………5  Post graduate degree…  முதுநிலைப் பட்டப்படிப்பு…... 6  No response……8  பதில் இல்லை | | |  | | --- | |  |
| C7 | Which of the following best describes your main work status/ occupation  over the past 12 months?  கடந்த 12 மாதங்களில் உங்கள் முக்கிய வேலை/ தொழிலை பின்வரும் எது சிறப்பாக விவரிக்கிறது?  If more than one, choose whichever was for the longer period in last 12 months. If not sure where the response would fit in, please record verbatim and code it later after clarification.  ஒன்றுக்கும் அதிகமாக இருந்தால். கடந்த 12 மாதங்களில் நீண்ட காலம் செய்த வேலையைப் பதிவு செய்யவும், பதில் எங்கே வரும் என்பது உறுதியாக தெரியவில்லை என்றால், வார்த்தை மாறாமல் பதிவு செய்யவும் மற்றும் தெளிவுபடுத்திய பின்னர் குறியிடவும் | Professional…….01தொழிற்படிப்பு படித்து தொழில் செய்பவர்Medium to large Business….02நடுத்தரம் முதல் பெரிய அளவிலான தொழில்Middle / Senior Executive/officer in organization…03நிறுவனத்தில் நடுநிலை / மேல்நிலை எக்சிக்யூடிவ் / அலுவலர்Agriculture land owner………….…….04விவசாய நில உரிமையாளர்Sales and Marketing executives/ Clerical...................05சேல்ஸ் மற்றும் மார்கெட்டிங் எக்சிக்யூடிவ்கள் / க்ளார்க் வேலைSelf-employed and small business ……...06சுய தொழில் மற்றும் சிறிய தொழில்Skilled manual laborer…….………11பயிற்சி பெற்று உடல் உழைப்பு சார்ந்த வேலை செய்பவர்Unskilled manual/agricultural laborer...12பயிற்சி பெறாமல் உடல் உழைப்பு சார்ந்த வேலை / விவசாயக் கூலி Student………………… 13  மாணவர்  Homemaker……………. 14  இல்லத்தரசி  Retired ………………15  ஓய்வுபெற்றவர்  Unemployed (able to work) …………16  வேலை இல்லாதவர் (வேலை செய்யக்கூடியவர்)  Unemployed (unable to work) ….17  வேலை இல்லாதவர் (வேலை செய்ய முடியாதவர்) Not in the list, then specify………….18பட்டியலில் இல்லை (குறிப்பிடுக)No response …..88பதில் இல்லை | | |  |  | | --- | --- |   If fills 18 write down verbatim:  18 குறிக்கப்பட்டால் எழதவும்  ____________________________________________ |  |
| C8 | Whether the individual’s house is in urban or rural?  *(Select as observed)*  பங்கேற்பாளரின் வீடு எங்கு உள்ளது?  (கிராமப்புறம் அல்லது நகர்ப்புறம்)  (கவனிக்கப்பட்டபடி எழுதவும்) | Urban…1 நகர்ப்புறம் Rural…2 கிராமப்புறம் | | |  | | --- | |  |
| C9 | In which type of area the individual’s household is? பங்கேற்பாளரின் வீடு என்ன வகையான பகுதியில் உள்ளது என கவனிக்கவும்? *(Select as observed)*  (கவனிக்கப்பட்டபடி எழுதவும்) | Slum…1குடிசைப் பகுதிNon-Slum…2குடிசைப் பகுதியற்றவை | | |  | | --- | |  |
| C10 | In which type of terrain, the individual’s household is? பங்கேற்பாளரின் வீடு எந்த வகையான நிலப்பரப்பில் உள்ளது? *(Select as observed)*  (கவனிக்கப்பட்டபடி எழுதவும்) | Hilly…1மலை சார்ந்த பகுதிPlain….2சமவெளி பகுதி | | |  | | --- | |  |
| C11 | Select the individual household type?  பங்கேற்பாளரின் வீடு எந்த வகையை சார்ந்தது?  *(Select as observed based on roof, floor and walls)*  (கவனிக்கப்பட்டபடி எழுதவும்) | Pucca…1நிலையானSemi-Pucca…2பாதி நிலையானKacha…3நிலையற்ற | | |  | | --- | |  |
| C12 | Do you have Chief minister’s comprehensive health insurance scheme card/ any Government insurance scheme card?  உங்களிடம் முதலமைச்சரின் விரிவான சுகாதார காப்பீட்டு திட்டத்தின் அட்டை அல்லது பிற அரசு சுகாதார காப்பீட்டு திட்டத்தின் அட்டை உள்ளதா? | Yes….1ஆம்No……2இல்லை | | |  | | --- | | *If yes go to T1*  ஆம் *என்றால் T1 செல்லவும* |
| C12 a | Are you eligible for Chief minister’s comprehensive health insurance scheme/ any Government health insurance scheme?  நீங்கள் முதலமைச்சரின் விரிவான சுகாதார காப்பீட்டு திட்டம் அல்லது பிற அரசு சுகாதார காப்பீட்டு திட்டத்திற்கு தகுதி உடையவரே? | Yes….1ஆம்No……2இல்லை | | |  | | --- | |  |

| **BEHAVIOURAL MEASUREMENTS** | | | | | |
| --- | --- | --- | --- | --- | --- |
| **Smoking/Tobacco use:**  **புகைப்பது - புகையிலை உபயோகிப்பது:** | | | | | |
| Now I am going to ask you some questions about various health related behaviors. We have to ask these questions as written, so please do not take offense as none is intended. This will include questions on smoking, drinking alcohol, eating fruits and vegetables and physical activity. Let’s start with tobacco  இப்போது, வெவ்வேறு ஆரோக்கியம் தொடர்பான பழக்கங்கள் குறித்த சில கேள்விகளை நான் உங்களிடம் கேட்கப் போகிறேன், எழுதியிருக்கும் படி நாங்கள் உங்களிடம் இந்த கேள்விகளைக் கேட்க வேண்டும். எதையும் தவறாக எடுத்துக் கொள்ள வேண்டாம், ஏனென்றால், உங்களைக் காயப்படுத்துவதற்கான எண்ணம் இல்லை. இவை புகைபிடிப்பது, மதுபானம் குடிப்பது, பழங்கள் மற்றும் காய்கறிகள் சாப்பிடுவது மற்றும் உடல் நடவடிக்கை மீதான கேள்விகளை உள்ளடக்கும். நாம் புகையிலையுடன் தொடங்கலாம் | | | | | |
| **Questions and Filter**  கேள்விகள் மற்றும் ஃபில்டர் | | **Response**  **பதில்** | | **Skip**  விட்டு விடவும் | |
| T1 | Do you **currently** smoke any **tobacco** products, such as bidis, cigarettes, cigars or pipes, hookah or any other local smoked tobacco products?  *(Use show card)*  *பீடிகள், சிகரெட்டுகள், சிகார்கள் அல்லது பைப்புகள் .* ஹூகா *அல்லது உள்ளுரில் புகைக்கப்படும் வேறு ஏதாவது புகையிலை பொருட்கள் எதையாவது நீங்கள் தற்போது புகைத்திருக்கிறீர்களா*?  (*கார்டு உபயோகிக்கவும்)*  Ask the participant to think of any tobacco products he/she is smoking currently  *பங்கேற்பாளரை அவர் தற்போது புகைத்துக்கொண்டிருக்கும் ஏதாவது புகையிலை பொருட்களைப் நினைத்துப் பார்க்கச் சொல்லவும்* | Yes....1  ஆம்  No………2  இல்லை | |  | | --- | | *If No, go to T6*  இல்லை என்றால். T6க்கு செல்லவும் | |
| T2 | Do you smoke tobacco products **daily**?  நீங்கள் தினசரி புகையிலை பொருட்களைப் புகைக்கிறீர்களா ?  This question is only for current smokers of tobacco products. Emphasize DAILY  புகையிலை பொருட்களைத் தற்போது பிடிப்பவர்களுக்கு மட்டுமே இந்த கேள்வி பொருந்தும். தினசரி என்பதை அழுத்திச் சொல்லவும், | Yes …….1  ஆம்  No……2  இல்லை | |  | | --- | |  | |
| T3 | On an average, **how many** (number of times in case of hookah) of the following products do you smoke **each day/week**?  சராசரியாக, **ஒவ்வொரு நாளும் / வாரமும்** பின்வரும் பொருட்களில் நீங்கள் எதனை **எத்தனை முறை**  (ஹூகா என்றால் முறைகளின் எண்ணிக்கை) புகைக்கிறீர்கள் ?   - *Record for each type, Use Show Card*   *ஒவ்வொரு வகையையும் பதிவு செய்யவும், கார்டை உபயோகிக்கவும்*   - *Record 0, if any product is not used instead of leaving blank in the products categories*.   ஏதாவது பொருளை உபயோகிப்பதில்லை என்றால், பொருள் பிரிவுகளில் காலியாக விடுவதற்கு பதிலாக 00 பதிவு செய்யவும்   - *Record for any new form of tobacco use reported by the Respondent but interpret the answers carefully e.g .if someone reports reverse smoking bidi it relates to a different method of smoking but the product remains bidi and should be marked as such.*   *குறிப்பிட்ட ஏதாவது புதிய வடிவ புகையிலையை உபயோகிக்கின்றனர் என்றால் உபயோகத்தினை பதிவு செய்யவும். ஆனால் பதிலை கவனிக்கவும் உதாரணமாக சிலர் திரும்பவும் பீடீ புகைகஙகின்றார் என்றால் பீடயில் தான் குறிக்க வேண்டும்*  *If less than daily, record weekly.* ***Fill only one of the two columns for each option****.*  *Enter '99' if respondent does not smoke bidis daily.*  தினசரியை விட குறைவு என்றால், வாரந்தோறும் பதிவு செய்யவும். ஒவ்வொரு வாய்ப்பிற்கும் இரண்டு கட்டங்களில் ஒன்றை மட்டும் பூர்த்தி செய்யவும், அடுத்த கட்டத்தில் 99 குறிப்படவும்  *If Don’t Know 77*  *தெரியாது என்றால் 77* Record daily consumption for daily smokers. If some products are smoked less than daily by daily smokers (for example a daily cigarette smoker may use bidi occasionally), enter weekly consumption. Also enter weekly consumption for current, non-daily smokers. remember that number of sticks or times (in case of Hokkah) need to be mentioned and not number of days  *தற்போது புகைப்பிடிப்பவர்களுக்கு மட்டும். தினசரி புகைப்பிடிப்பவர்களுக்கு தினசரி பிடிப்பதைப் பதிவு செய்யவும், தினசரி புகைப்பிடிப்பவர்கள் சில பொருட்களை குறைவாக பிடித்தால் (உதாரணமாக தினசரி சிகரெட் பிடிப்பவர் எப்போதாவது பிடி பீடிக்கலாம்). வாரந்தோறும் பிடிப்பதில் பதிவு செய்யவும், மேலும் தற்போது புகைப்பிடிப்பவர்கள், தினசரி புகைப்பிடிப்பவர்களுக்க வாரந்தோறும் பிடிப்பதையும் பதிவு செய்யவும்.* | | | | |
|  |  | Daily Frequency  தினசரி கால இடைவெளி | Weekly Frequency  வாரந்தோறும் கால இடைவெளி | |  |
| T3a | Bidis  பீடிககள் | |  |  | | --- | --- | | |  |  |  | | --- | --- | --- | | |  |
| T3b | Manufactured Cigarettes  தயாரிக்கப்பட்ட சிகரெட்டுகள் | |  |  | | --- | --- | | |  |  |  | | --- | --- | --- | | |  |
| T3c | Hand-rolled Cigarettes  கைகளால்-சுருட்டப்பட்ட சிகரெட்டுகள் | |  |  | | --- | --- | | |  |  |  | | --- | --- | --- | | |  |
| T3d | Pipes  பைப்புகள் | |  |  | | --- | --- | | |  |  |  | | --- | --- | --- | | |  |
| T3e | Cigars, Cheroots  சிகார்கள், சுருட்டுகள் | |  |  | | --- | --- | | |  |  |  | | --- | --- | --- | | |  |
| T3f | Hookah/No. of Shisha session  ஹூகா – ஷிஷா புகைக்கும் சந்தர்பங்களின் எண்ணிக்கை | |  |  | | --- | --- | | |  |  |  | | --- | --- | --- | | |  |
| T3g | E- cigarette  இ-சிகரெட் | |  |  | | --- | --- | | |  |  |  | | --- | --- | --- | | |  |
| T3h | Other local **smoked** tobacco products  உள்ளுரில் புகைக்கப்படும் மற்ற புகையிலைப் பொருட்கள் | |  |  | | --- | --- | | |  |  |  | | --- | --- | --- | | |  |
| T3i | Other (Please Specify)  (Enter 99 if respondent does not smoke any other type of tobacco products)  மற்றவை (தயவு செய்து குறிப்பிடவும்)  …………………………………… | |  |  | | --- | --- | | |  |  |  | | --- | --- | --- | | |  |
| T4 | During the past 12 months, have you tried **to stop smoking**  கடந்த 12 மாதங்களில், நீங்கள் புகைப்பதை நிறுத்துவதற்கு முயற்சி செய்திருக்கிறீர்களா?  For current smokers only. Ask the participant to think of any quit attempt they made during the past 12 months. This hasto be a real attempt and not just a thought.  *Please validate your data!*  தற்போது புகைப் பிடிப்பவர்களுக்கு மட்டும், பங்கேற்பாளரை கடந்த 12 மாதங்களில் புகைப்பிடிப்பதை நிறுத்துவதற்கு மேற்கொள்ளப்பட்ட ஏதாவது முயற்சியை நினைத்துப் பார்க்கச் சொல்லவும். | Yes----------1  ஆம்  No……….2  இல்லை | |  | | --- | |  | |
| T5 | During any visit to a doctor or other health worker in the past 12 months, were you advised to quit smoking tobacco?  கடந்த 12 மாதங்களில், ஒரு மருத்துவர் அல்லது மருத்துவ ஊழியரிடம் சென்ற போது, புகையிலை பிடிப்பதை விடும்படி உங்களுக்கு அறிவுரை அளிக்கப்பட்டதா?  Ask the participant to think of visits to a doctor or other health worker during the past 12 months. If no visit, select “no visit during the past 12 months”. தற்போது  பங்கேற்பாளரை ,கடந்த 12 மாதங்களில் ஒரு மருத்துவர் அல்லது மருத்து ஊழியரைச் சந்திக்கச் சென்றதை நினைத்துப் பார்க்கச் சொல்லவும், செல்லவில்லை என்றால். “கடந்த 12 மாதங்களில் சந்திக்க செல்லவில்லை்” என்பதை தேர்ந்தெடுக்கவும | Yes----------1  ஆம்  No….2  இல்லை No visit in the  last 12 months period.............3  கடந்த 12 மாதங்களில் செல்லவில்லை | |  | | --- | |  | |
| T6 | In the **past**, did you **ever** smoke any tobacco products?  (*Use show card)*  ***கடந்த காலத்தில்****, நீங்கள்* ***எப்போதாவது*** *ஏதாவது புகையிலை பொருட்களைப் புகைத்தீர்களா* ?  (*கார்டை உபயோகிக்கவும்)*  Ask the participant to think of the time when he/she may have been smoking tobacco products.  பங்கேற்பாளர் புகையிலை பொருட்களை புகைத்துக் கொண்டிருந்த காலத்தை நினைத்துப் பார்க்கச் சொல்லவும். | Yes ….1  ஆம்  No ….2  இல்லை | |  | | --- | | *If No, go to* T11  இல்லை *என்றால், T11க்கு செல்லவும்* | |
| T7 | In the past, did you **ever** smoke **daily**?  கடந்த காலத்தில், நீங்கள் **எப்போதாவது** **தினசரி** புகைப்பிடித்தீர்களா?  Ask the participant to think of the time when he/she may have been smoking tobacco products on a daily basis.  தினசரி அடிப்படையில் அவர் புகையிலை பொருட்களைப் புகைத்துக் கொண்டிருந்த காலத்தை நினைத்துப் பார்க்கச் சொல்லவும். | Yes ….1  ஆம்  No ….2  இல்லை | |  | | --- | |  | |
| T8 | How old were you when you **first** started smoking?  நீங்கள் புகைக்கத் **தொடங்கிய** போது உங்கள் வயது என்ன?  Ask the participant to think of the time when he/she started to smoke any tobacco products.  தற்போது புகைப்பவர்களுக்கு மட்டும். பங்கேற்பாளரை அவர் ஏதாவது புகையிலை பொருட்களைப் தினசரி புகைக்கத் தொடங்கிய காலத்தை நினைத்துப் பார்க்கச்சொல்லவும்.. | Age in completed years  பூர்த்தியடைந்த வருடங்களில் வயது  Don’t remember…77  ஞாபகமில்லை | |  |  | | --- | --- | |  | |
| T9 | How old were you when you **stopped** smoking?  நீங்கள் புகைப்பதை **நிறுத்திய** போது உங்கள் வயது என்ன?  Ask the participant to think of the time when he/she stopped  smoking tobacco products.  பங்கேற்பாளர் புகையிலை பொருட்களை நிறுத்திய காலத்தை நினைத்துப் பார்க்கச் சொல்லவும் | Age in completed years  பூர்த்தியடைந்த வருடங்களில் வயது  Don’t remember….77  ஞாபகமில்லை | |  |  | | --- | --- | | *If provided go T11 பதில் இருந்தால் T11க்கு செல்லவும்* | |
| T10 | How **long ago** did you stop smoking?  நீங்கள் எவ்வளவு காலத்திற்கு முன் புகைப்பதை நிறுத்தினீர்கள்?  If the participant doesn't remember his/her age when they stopped smoking, then record the time in years as appropriate  Leave blank if not known, otherwise, answer must be between 1 and 61 Years.  அவர்கள் புகைப்பதை நிறுத்திய போது, பங்கேற்பாளருக்கு அவருடைய வயது ஞாபகமில்லை என்றால், பொருந்துவதற்கு ஏற்ப வாரங்கள், மாதங்கள் அல்லது வருடங்களைப் பதிவு செய்யவும் | In Years  வருடங்களில்  Don’t remember…77  ஞாபகமில்லை | |  |  | | --- | --- | |  | |

| **Smokeless Tobacco use**  **புகையில்லாத புகையிலை உபயோகம்** | | | | | | | | | | | |
| --- | --- | --- | --- | --- | --- | --- | --- | --- | --- | --- | --- |
| **Now I shall ask you about smokeless tobacco like** chewing tobacco, snuff, tobacco containing betel, gutka, pan masala, etc**.**  இப்போது புகையிலையை மெல்வது, மூக்கு பொடி, பாக்கு அடங்கிய புகையிலை, குட்கா, பான் மசாலா போன்ற புகையில்லாத புகையிலைப் பற்றி நான் உங்களிடம் பேசுவேன். | | | | | | | | | | | |
| **Questions** | | | | **Response** | | | | **Skip** | | | |
| T11 | Do you **currently use** any **smokeless tobacco**, such as chewing tobacco, tobacco containing betel, gutka, pan masala, etc.?  [Use showcard]  புகையிலையை மெல்வது, மூக்கு பொடி, பாக்கு அடங்கிய புகையிலை, குட்கா, பான் மசாலா போன்ற ஏதாவது **புகையில்லாத புகையிலையை** நீங்கள் **தற்போது உபயோகிக்கிறீர்களா?**  Ask the participant to think of any smokeless tobaccoproducts that he/she is using currently.  பங்கேற்பாளரை அவர் தற்போது உபயோகித்துக் கொண்டிருக்கும் புகையிலை பொருட்களை நினைத்துப் பார்க்கச் சொல்லவும் | | | Yes ….1  ஆம்  No ….2  இல்லை | | |  | | --- | | | *If No, go to T14*  *இல்லை என்றால், T14க்கு செல்லவும்* | | | |
| T12 | Do you **currently use smokeless tobacco** products **daily?**  **ஆம் என்றால், நீங்கள் தற்போது தினசரி புகையில்லாத புகையிலை பொருட்களை உபயோகிக்கிறீர்களா?**  For current users of smokeless tobacco products only.  தற்போது புகையில்லாத புகையிலை பொருட்களை மட்டும் உபயோகிப்பவர்களுக்கு | | | Yes ….1  ஆம்  No ….2  இல்லை | | |  | | --- | | |  | | | |
| T13 | On average, how many **times a day/week** do you use?  சராசரியாக, ஒரு நாளில் / வாரத்தில் எத்தனை முறை நீங்கள் …………… உபயோகிக்கிறீர்கள்   - *Record for each type, Use Show Card*   *ஒவ்வொரு வகைக்கும் பதிவு செய்யவும், கார்டை உபயோகிக்கவும்*   - *Record 00, if any product is not used instead of leaving blank in the products categories.*   *ஏதாவது பொருட்கள் உபயோகிக்கப்படுவதில்லை என்றால் பொருட்கள் பிரிவுகளில் காலியாக விடுவதற்கு பதிலாக 00 பதிவு செய்யவும்*  *If less than daily, record weekly.* ***Fill only one of the two columns for each option****. The other column would be then marked 99*  தினச*ரி*யை விட குறைவு என்*றா*ல், வாரந்தோறும் பதிவு செய்யவும், ஒவ்வொரு வாய்ப்பிற்கும் இரண்டு கட்ட*ங்*களில் ஒன்றை மட்டும் பூர்த்தி செய்யவும், அடுத்த கட்டத்தில் 99 குறிப்படவும்  *Don’t Know 77 தெரியாது77*  Record for each type of smokeless tobacco products. daily consumption for daily users. If products are used less than daily by daily users, For example a daily tobacco chewer may use Betel leaves occasionally, then enter weekly consumption. Also enter weekly consumption for current, non-daily users.  ஒவ்வொரு வகையான புகையில்லாத புகையிலை பொருட்களுக்கும் பதிவு செய்யவும். தினசரி உபயோகிப்பவர்கள், தினசரியை விட குறைவாக உபயோகித்தால், உதாரணமாக தினசரி புகையிலை மெல்பவர் எப்போதாவது வெற்றிலைகளை உபயோகிக்ககூடும். அப்படியானால் வாரந்தோறும் சாப்பிடுவதை டைப் செய்யவும். மேலும் தற்போது , தினசரி உபயோகிக்காதவர்களுக்கு வாரந்தோறும் சாப்பிடுவதை டைப் செய்யவும். | | | | | | | | | | |
|  |  | | Daily frequency  தினசரி கால  இடைவெளி | | | | Weekly frequency  வாரந்தோறும் கால  இடைவெளி | | |  | |
| T13a | Chewing tobacco  புகையிலை மெல்வது | | |  |  | | --- | --- | | | | | |  |  | | --- | --- | | | | *Go to T16*  *T16க்கு செல்லவும்* | |
| T13b | Pan with zarda, Betel with Tobacco, quid  ஜர்தாவுடன் வெற்றிலை , புகையிலையுடன் பாக்கு | | |  |  | | --- | --- | | | | | |  |  | | --- | --- | | | |
| T13c | Tobacco Snuff, by mouth  புகையிலைப் பொடி வாய் வழியாக எடுத்துக் கொள்வது | | |  |  | | --- | --- | | | | | |  |  | | --- | --- | | | |
| T13d | Snuff, by nose  மூக்குப் பொடி. மூக்கு வழியாக | | |  |  | | --- | --- | | | | | |  |  | | --- | --- | | | |
| T13e | Any other smokeless tobacco. Please specify other types of tobacco used.  மற்றவை (குறிப்பிடவும்)  ……………………………….. | | |  |  | | --- | --- | | | | | |  |  | | --- | --- | | | |
| T14 | In the **past** did you **ever use** smokeless tobacco products such as chewing tobacco, tuibu, snuff, betel, gutaka, etc.?  **கடந்தகாலத்தில்** மெல்வதற்கான புகையிலை, பொடி, பாக்கு, குட்கா போன்ற புகையில்லாத புகையிலைப் பொருட்களை நீங்கள் **எப்போதாவது உபயோகித்தீர்களா**?  Ask the participant to think of the time when he/she may have been using smokeless tobacco products  பங்கேற்பாளரை அவர் புகையில்லாத புகையிலைப் பொருட்களை உபயோகித்திருக்ககூடிய காலத்தை நினைத்துப் பார்க்கச் சொல்லவும் | | Yes ….1  ஆம்  No ….2  இல்லை | | | | |  | | --- | | | | *If No go to T18*  *இல்லை என்றால், T18க்கு செல்லவும்* | |
| T15 | In the **pas**t, did you **ever** use smokeless tobacco products such as [snuff, chewing tobacco, or betel] **daily**?  **கடந்த காலத்தில்**, பொடி, மெல்வதற்கான புகையிலை அல்லது பாக்கு போன்ற புகையில்லாத புகையிலை பொருட்களை நீங்கள் **எப்போதாவது தினசரி** உபயோகித்தீர்களா ?  Ask the participant to think of the time when he/she may have been using smokeless tobacco products on a daily basis.  பங்கேற்பாளர் புகையில்லாத புகையிலைப் பொருட்களை தினசரி அடிப்படையில் உபயோகித்திருக்ககூடிய காலத்தை நினைத்துப் பார்க்கச் சொல்லவும் | | Yes ….1  ஆம்  No ….2  இல்லை | | | | |  | | --- | | | |  | |
| T16 | How old were you at that time when you **first started** using smokeless tobacco?  நீங்கள் புகையில்லாத புகையிலையை முதலில் உபயோகிக்கத் **தொடங்கிய போது** உங்கள் வயது என்ன?  Ask the participant to think of the time when he/she started to using any smokeless tobacco  products.  புகையில்லாத புகையிலை பொருட்கள் எதையாவது உபயோகிப்பதற்கு அவர் தொடங்கிய காலத்தை பங்கேற்பாளரை நினைத்துப் பார்க்கச் சொல்லவும் | | Age in completed years  முடிவடைந்த வருடங்களில்  Don’t remember  ஞாபகமில்லை…77 | | | | |  |  | | --- | --- | | | |  | |
| T17 | How old were you when you **stopped** taking smokeless tobacco products?  நீஙகள் புகையில்லாத புகையிலை பொருட்களை **நிறுத்திய**போது உங்கள் வயது என்ன?  Ask the participant to think of the time when he/she stopped using smokeless tobacco products.  Can ask to correlate with special incidents in the person life or major well known events  அவர் புகைக்காத புகையிலை பொருட்களை நிறுத்திய சமயத்தை பங்கேற்பாளரை நினைத்துப் பார்க்கச் சொல்லவும், தனிப்பட்ட வாழ்க்கையில் அல்லது பெரிய, நன்கு அறியப்பட்ட நிகழ்வுகளுடன் இணைத்து யோசிக்கச் சொல்லலாம் | | Age in completed years  முடிவடைந்த வருடங்களில்  Don’t remember ஞாபகமில்லை…77 | | | | |  |  | | --- | --- | | | |  | |
|  |  | |  | | | |  | | |  | |
| **Passive Smoking** | | | | | | | | | | | |
| Now I shall ask you about exposure to smoke because of smokers near you who share room/ space with you even if you are not smoking  நீங்கள் புகைக்கவில்லை என்றாலும்; உங்களுடன் அறை / இடத்தைப் பகிர்ந்து கொள்ளும் நபர்கள் புகைப்பதனால் செயலற்ற புகைத்தல் பற்றி நான் உங்களிடம் கேட்பேன். | | | | | | | | | | | |
| T18 | | During the past 30 days, did someone smoke in your home when you were present? (including any visitors)  கடந்த 30 நாட்களில், நீங்கள் வீட்டில் இருந்த போது யாராவது விருந்தினர்கள் உட்பட வீட்டிற்குள் புகைபிடித்தார்களா?  The participant should only think about other people, not about him-/herself. Smokers should exclude themselves.  பங்கேற்பாளர் மற்றவர்களைப் பற்றி மட்டுமே நினைக்க வேண்டும் , அவரைப் பற்றி நினைக்க கூடாது. புகைப்பவர்கள் தங்களைச் சேர்த்துக் கொள்ளக்கூடாது.  The question is asking about inside the participant’s home. This only includes fully enclosed areas of the home.  இந்த கேள்வி பங்கேற்பாளரின் வீட்டின் உள்ளே இருப்பதைப் பற்றி கேட்கிறது. இது வீட்டில் முழுமையாக மூடப்பட்ட பகுதியை மட்டுமே உள்ளடக்குகிறது. | | | Yes……….  ஆம் ……..1  No……….  இல்லை ……2  Do not remember-----7 ஞாபகமில்லை  No response/ refuse….8  பதில் இல்லை / மறுத்தல் | | | | |  | | --- | | |  |
| T19 | | During the past 30 days, did someone smoke in closed areas in your workplace (in the building, in a work area or a specific office) in your presence?  கடந்த 30 நாட்களில், நீங்கள் வேலை செய்யும் இடத்தில் நீங்கள் இருந்த போது மூடப்பட்டிருந்த பகுதிகளில் யாராவது புகைத்தார்களா (கட்டிடத்தில் , வேலை செய்யும் இடத்தில், குறிப்பிட்ட அலுவலகத்தில்)?  For those not working in a closed area, record “don’t work ina closed area”.  மூடப்பட்ட பகுதியில் வேலை செய்யாதவர்களுக்கு, ”மூடப்பட்ட பகுதியில் வேலை செய்யவில்லை்” என்பதைப் பதிவு செய்யவும்  Ask the participant to think of seeing somebody smoke or smelling the smoke in indoor areas at work during the past 30 days.  பங்கேற்பாளரை, கடந்த 30 நாட்களாக வேலையின் போது உட்புற பகுதிகளில் ஒருவர் புகைத்ததை பார்த்தது அல்லது புகையை முகர்ந்ததை நினைத்துப் பார்க்கச் சொல்லவும். | | | Yes ஆம் …….1  No இல்லை … …2  Do not know……7  தெரியாது  Do not work in closed areas….3  மூடப்பட்ட பகுதியில் வேலை செய்யவில்லை  Do not go out for work….4 வேலை செய்ய வெளியில் செல்லவில்லை.  No response/ refuse….8  பதில் இல்லை / மறுத்தல் | | | | |  | | --- | | |  |
| T20 | | During the past 30 days, did someone smoke while you were travelling in car/ bus/ train/metro etc.?  கடந்த 30 நாட்களில், நீங்கள் கார் - பஸ் - ரயில் - மெட்ரோ போன்றவற்றில் பயணம் செய்த போது யாராவது உங்கள் அருகில் புகைபிடித்தார்களா?  Ask the participant to think of seeing somebody smoke or smelling the smoke in a vehicle while travelling during the past 30 days.  கடந்த 30 நாட்களில் பயணம் செய்யும் போது, ஒருவர் புகைத்ததைப் பார்த்தது அல்லது வண்டியில் புகையின் மணத்தை உணர்ந்ததை பங்கேற்பாளரை நினைத்துப் பார்க்கச் சொல்லவும். | | | Yes ஆம் …….1  No இல்லை… …2  Do not remember-----7 ஞாபகமில்லை  No response/ refuse….8  பதில் இல்லை / மறுத்தல்  Did not travel in a vehicle the last 30 days….9  கடந்த 30 நாட்களில் ஒரு வண்டியில் பயணம் செய்யவில்லை | | | | |  | | --- | | |  |

| **Alcohol Consumption மதுபானம் குடிப்பது** | | | | | | | |
| --- | --- | --- | --- | --- | --- | --- | --- |
| The next questions relate to consumption of alcohol. As already said please do not get offended and answer truthfully as this information is confidential  அடுத்த சில கேள்விகள் மதுபானம் குடிப்பது தொடர்பானது, முன்பே சொன்னது போல் சங்கடப்படவேண்டாம், உண்மையை சொல்லவும். இந்த விபரம் ரகசியமாக வைக்கப்படும். | | | | | | | |
| **Questions**  **கேள்விகள்** | | **Responses**  **பதில்கள்** | | | | **Skip** | |
| A1 | Have you **ever** consumed any alcoholic products such as beer, wine, whisky, locally prepared alcohol, etc.?  நீங்கள் **எப்போதாவது** ஏதாவது மதுபானப் பொருட்களைச் குடித்திருக்கீர்களா? (பீர், வைன், விஸ்கி, உள்ளுரில் தயாரிக்கப்பட்ட மதுபானம் போன்றவை)  *(Use Showcard /show examples)*  கார்டை உபயோகிக்கவும் / உதாரணங்களைக் காண்பிக்கவும்  Ask the participant to think of any drink that contains alcohol, with the exception of alcohol-based medication that is taken due to health reasons or alcohol consumed for religious reasons.  பங்கேற்பாளரை மதுபானம் அடங்கிய ஏதாவது பானத்தை நினைத்துப் பார்க்கச் சொல்லவும், உடல் நலம் பெறுவதற்கான காரணங்களுக்காக எடுத்துக் கொள்ளப்படும் அல்லது மதம் சார்ந்த காரணங்களுக்காக குடிக்கப்படும் மதுபானம் - அடிப்படையிலானவற்றைச் சேர்க்க வேண்டாம் | Yes ….1  ஆம்  No ….2  இல்லை | | |  | | --- | | | *If No, go to D1*  *இல்லை என்றால். D1க்கு செல்லவும்* | |
| A2 | How **old** were you when you **first** consumed alcohol?  நீங்கள் **முதலில்** மதுபானம் குடித்த போது உங்கள் **வயது** என்ன?  Ask the participant to think of the age when he/she started consuming alcohol  பங்கேற்பாளரை அவர் மதுபானம் குடிக்கத் தொடங்கிய வயதை நினைத்துப் பார்க்கச் சொல்லவும் | Age in completed years  முடிவடைந்த வருடங்களில்  Don’t remember  ஞாபகமில்லை…77 | | |  |  | | --- | --- | | |  | |
| A3 | Have you consumed any alcoholic products within the **past 12months**?  **கடந்த 12 மாத**ங்களுக்குள் நீங்கள் ஏதாவது மதுபானப் பொருட்களைக் குடித்திருக்கிறீர்களா?  Ask the participant to think of any drinks that contain alcohol, with the exception of alcohol-based medication that is taken due to health reasons or for religious reasons in the last 12 months  பங்கேற்பாளரை கடந்த மாதங்களில் குடித்த மதுபானம் அடங்கிய ஏதாவது பானத்தை நினைத்துப் பார்க்கச் சொல்லவும், உடல் நலம் பெறுவதற்கான காரணங்களுக்காக எடுத்துக் கொள்ளப்படும் அல்லது மதம் சார்ந்த காரணங்களுக்காக குடிக்கப்படும் மதுபானம் - அடிப்படையிலானவற்றைச் சேர்க்க வேண்டாம் | Yes ….1  ஆம்  No ….2 இல்லை | | |  | | --- | | | *If No, go to A12 இல்லை என்றால். A16க்கு செல்லவும்* | |
| Spend some time trying to understand the alcohol use pattern of the respondent and estimate most appropriate standard drink size  பதிலளிப்பவர் அதிக சராசரி அளவை சரியாக புரிந்து கொண்டார் என தெரிந்து கொள்ள சற்று நேரம் செலவிடவும்  Use this space to get your estimates: உங்களின் தோராய அளவை அறிய இந்த இடத்தை பயன்படுத்தவும்  Commonly consumed alcoholic drink ____________ alcohol concentration (%) ______( standard drink size)______  பொதுவாக உட்கொள்ளும் மது மது கலந்தது நிலையான பானம்  Commonly consumed alcoholic drink ____________ alcohol concentration (%) ______( standard drink size)______  பொதுவாக உட்கொள்ளும் மது மது கலந்தது நிலையான பானம்  Commonly consumed alcoholic drink ____________ alcohol concentration (%) ______( standard drink size)______  பொதுவாக உட்கொள்ளும் மது மது கலந்தது நிலையான பானம் | | | | | | | |
| A4 | In past 12 months, **how frequently** have you had at least one standard alcoholic drink (clarify one standard drink to the respondent using the calculations done above)?  கடந்த 12 மாதங்களில், நீங்கள் குறைந்தபட்சம் குறைந்தபட்சம் ஒரு நிலையான அளவு மதுபானத்தை எவ்வளவு கால இடைவெளியில் **அடிக்கடி** குடித்திருக்கிறீர்கள் ?   - *Read Responses* - *பதில்களை வாசிக்கவும்* - For those that have consumed alcohol in the past 12 months. A “standard drink” is the amount of ethanol contained in standard glasses of beer, wine, fortified wine such as sherry, and spirits.   (*Use showcard)*   - *கடந்த 12 மாதங்களில் மதுபானம் குடித்திருப்பவர்களுக்கு . ஒரு ”நிலையான அளவு பானம் என்றால்*, *பீர்*, *வைன்* ,*ஷெரி மற்றும் ஸ்பிரிட்ஸ் போன்ற செரிவூட்டப்பட்ட வைனில் நிலையான அளவு க்ளாசில் அடங்கியிருக்கும் எ*த்*தனால் அளவாகும்.* | | Daily... 1  தினசரி  5-6 days per week ...2  ஒரு வாரத்தில் 5-6 நாட்கள்  3-4 days per week ...3  ஒரு வாரத்தில் 3-4 நாட்கள்  1-2 days per week …4  ஒரு வாரத்தில் 1-2 நாட்கள்  1-4 days per month …5  ஒரு மாதத்தில் 1-4 நாட்கள்  Less than once a month….6  ஒரு மாத்த்திற்கு ஒரு முறையை விட குறைவாக | | |  | | --- | | |  |
| A5 | During the past 12 months, how often have you **needed** **a first drink** in the morning to get yourself going after a heavy drinking session?  கடந்த 12 மாதங்களில், மிகஅதிக அளவு குடித்த தருணங்களில் , அடுத்த நாள் காலையில், எழுந்தவுடன் உங்கள் வேலைகளை செய்யவதற்கு எத்தனை முறை மதுபானம் தேவைப்பட்டது ?  Ask the participant to think of the past 12 months. Read out all the answer options. e.g. Out of 4 such occasions if he/she needed in 3 occasions then it has to be taken as often  பங்கேற்பாளரை கடந்த 12 மாதங்களைப் பற்றி நினைத்துப் பார்க்கச் சொல்லவும், பதிலுக்கான வாய்ப்புகள் அனைத்தையும் வாசிக்கவும். | Daily or almost daily தினந்தோறும் அல்லது பெரும்பாலும் தினசரி …...1  Weekly வாரந்தோறும்.... .2  Monthly மாதந்தோறும் …3  Less than monthly  ஒரு மாதத்திற்கும் குறைவாக….4  Never. ஒருபோதும் இல்லை. …5  Does not know … தெரியாது ..7  No response பதில் இல்லை ….8 | | | |  | | --- | | |  |
| A6 | Have you consumed any alcohol drink within the past **30 days**?  **கடந்த 30** நாட்களுக்குள் நீங்கள் ஏதாவது மதுபானத்தைக் குடித்திருக்கிறீர்களா?  Select the appropriate response  பொருத்தமான பதிலைத் தேர்ந்தெடுக்கவும் | Yes .….1  ஆம்  No ..….2  இல்லை | | | |  | | --- | | | *If No, go to D1*  *இல்லை என்றால். A16க்கு செல்லவும்* |
| A7 | During the past 30 days, on how many **occasions** did you have at least one standard alcoholic drink?  கடந்த 30 நாட்களில். எத்தனை **சந்தர்ப்பங்களில்** நீங்கள் குறைந்தபட்சம் ஒரு நிலையான அளவு மதுபானத்தைக் குடித்திருக்கிறீர்கள்?  Ask the participant to think of the past 30 days only. Record the number of occasions. Note that there can be more than one occasion in which alcohol is consumed in a given day.  பங்கேற்பாளரை கடந்த 30 நாட்களை மட்டும் நினைத்துப் பார்க்கச் சொல்லவும். சந்தர்ப்பங்களின் எண்ணிக்கையைப் பதிவு செய்யவும். ஒரு குறிப்பிட்ட நாளில் ஒன்றுக்கும் அதிகமான சந்தர்ப்பங்களின் மதுபானம் குடிக்கப்படலாம் என்பதை கவனத்தில் கொள்ளவும். | Number  எண்ணிக்கை  Don't know ……77  தெரியாது | | | |  |  | | --- | --- | | |  |
| A8 | During the past 30 days, when you drank alcohol, how many **standard drinks on average** did you have during one drinking occasion?  கடந்த 30 நாட்களில். நீங்கள் மதுபானம் குடித்த போது, குடிப்பதற்கான ஒரு சந்தர்ப்பத்தில் நீங்கள் **சராசரியாக** எத்தனை **நிலையான அளவு பானங்களைக்** குடித்தீர்கள்?   - *Use showcard*   *கார்டை உபயோகிக்கவும்*  Help the participant to average out the total number of drinks by using the showcard that shows standard alcoholic drinks  *நிலையான அளவு மதுபானங்களைக் காண்பிக்கும் கார்டை உபயோகிப்பதன் மூலமாக பானங்களின் சராசரியான மொத்த எண்ணிக்கையை கணக்கிடுவதற்கு பங்கேற்பாளருக்கு உதவவும்* | Number  எண்ணிக்கை  Don't know ……77  தெரியாது | | | |  |  | | --- | --- | | |  |
| A9 | During the past 30 days, what was the **largest number** of standard drinks you had on a single occasion, counting all types of alcoholic drinks together?  கடந்த 30 நாட்களில். அனைத்து வகையான மதுபானங்களையும் கணக்கில் எடுத்துக்கொள்ளும் போது, ஒரு சந்தர்பத்தில் நீங்கள் குடித்த நிலையான அளவு பானங்களின் **அதிகபட்ச எண்ணிக்கை** என்ன?  Ask the participant to think of the past 30 days only. This question is about the largest number of drinks that the participant had on one single occasion.  பங்கேற்பாளரை கடந்த 30 நாட்களை மட்டுமே நினைத்துப் பார்க்கச் சொல்லவும். இந்த கேள்வி ஒரு சந்தர்பத்தில், பங்கேற்பாளர் குடித்த பானங்களின் அதிகபட்ச எண்ணிக்கைப் பற்றியது. | Largest number  அதிகபட்ச எண்ணிக்கை  Don't Know…. 77  தெரியாது | | | |  |  | | --- | --- | | |  |
| A10 | During the past 30 days, how many times did you have **Six or more standard Drinks in a single drinking occasion?**  கடந்த 30 நாட்களில், எத்தனை முறை நீங்கள் ஆறு அல்லது அதிகமான நிலையான அளவு பானங்களைக் குடித்தீர்கள்?  Ask the participant to think of the past 30 days only, and to report the number of occasions when he/she had six or more standard drinks. Note that the question is NOT different for men and women.  பங்கேற்பாளரை கடந்த 30 நாட்களைப் பற்றி மட்டும் நினைத்துப் பார்த்து, அவர் ஆறு அல்லது அதிகமாக நிலையான அளவு பானங்களைக் குடித்த சந்தர்பங்களை தெரிவிக்கச் சொல்லவும். இந்த கேள்வி ஆண்கள் மற்றும் பெண்களுக்கு தனித்தனி அல்ல என்பதை கவனத்தில் கொள்ளவும். | Number of times  முறைகளின் எண்ணிக்கை  Don't Know.... 77  தெரியாது | | | |  |  | | --- | --- |   1 | |  |

**The next question will refer to your consumption of alcohol from sources other than authorised shop.**

| A11 | During the past 7 days did you consume home brewed alcohol/ illegally brewed alcohol?  *Use show card*  கடந்த 7 நாட்களில், நீங்கள் வீட்டில் வடிக்கப்பட்ட அல்லது சட்ட விரோதமாக தயாரிக்கப்பட்ட மதுபானத்தைக் குடித்தீர்களா? | Yes.…...1  ஆம்  No..…...2  இல்லை  Didn’t drink alcohol in last 7 days….9  கடந்த 7 நாட்களில், மது அருந்தவில்லை | | |  | | --- | | |  | |
| --- | --- | --- | --- | --- | --- | --- | --- | --- |
| A12 | Have you stopped drinking due to health reasons, such as a negative impact on your health or on the advice of your doctor or other health worker?  ஆரோக்கிய காரணங்களுக்காக அல்லது உங்கள் ஆரோக்கியத்தின் மீது பாதகமான தாக்கம் அல்லது உங்கள் மருத்துவர் அல்லது மற்ற மருத்துவ ஊழியரின் அறிவுரை போன்றவை காரணமாக நீங்கள் குடிப்பதை நிறுத்தியிருக்கிறீர்களா?  *This question is for those participants who have not taken alcohol during the past 12 months, but have taken at some time in their lifetime.*  இந்த கேள்வி கடந்த 12 மாதங்களில் குடித்திருக்காதவர்களுக்கு ஆனால் அவர்களுடைய வாழ்நாளில் எப்போதாவது குடித்திருப்பவர்களுக்கானது | | Yes.....1  ஆம்  No..…...2  இல்லை | | |  | | --- | | |  |

| Diet and cooking habits | | | | | | | | | | |
| --- | --- | --- | --- | --- | --- | --- | --- | --- | --- | --- |
| The next questions I will ask about the fruits and vegetables that you usually eat. I have a nutrition card here that shows you some examples of local fruits and vegetables. As you answer these questions please do not think only of the past one week (which might be different from what you usually do – like there might have been a family occasion last week or you could have been out vacationing last week) instead think of a ‘**typical’ or a ‘usual**’ week. Look at daily consumption by clubbing together various dishes/ sessions of eating  அடுத்த கேள்விகள், நீங்கள் வழக்கமாக சாப்பிடும் பழங்கள் மற்றும் காய்கறிகள் பற்றியது. உள்ளுர் பழங்கள் மற்றும் காய்கறிகளின் சில உதாரணங்களைக் காண்பிக்கும் ஒரு ஊட்டச்சத்து கார்டு என்னிடம் இருக்கிறது. நீங்கள் இந்த கேள்விகளுக்கு பதில் அளிக்கும் போது, கடந்த ஒரு வாரத்தை (இது நீங்கள் வழக்கமாக செய்வதை விட வித்தியாசமானதாக இருக்கலாம் - அதாவது கடந்த வாரம் ஒரு குடும்ப சந்தர்பம் இருந்திருக்கலாம் அல்லது கடந்த வாரம் நீங்கள் விடுமுறையில் சென்றிருக்கலாம்) மட்டும் நினைக்க வேண்டாம். அதற்கு பதிலாக ஒரு **‘வழக்கமான’** வாரத்தை நினைத்துப் பார்க்கவும் | | | | | | | | | | |
| **Questions**  **கேள்விகள்** | | | | **Response**  **பதில்கள்** | | | **Skip** | | | |
| D1 | In a typical week, on how many days do you **eat fruits**?  ஒரு வழக்கமான வாரத்தில், எத்தனை நாட்கள் நீங்கள் **பழ**ங்கள் **சாப்பிடுகிறீர்கள்?**  *(Use showcard)*  *(கார்டை உபயோகிக்கவும்)*    Ask the participant to think of any fruit on the showcard. A typical week means a "normal" week when the diet is not affected by cultural, religious, or other events. Ask the participant to not report an average over a period.  பங்கேற்பாளரை கார்டின் மீதுள்ள ஏதாவது பழத்தை நினைக்கச் சொல்லவும். ஒரு வழக்கமான வாரம் என்பது ஒரு “சாதாரணமான” வாரத்தை குறிப்பிடுகிறது, இதில், கலாச்சாரம், மதம் அல்லது மற்ற நிகழ்ச்சிகளால் உணவு பாதிக்கப்படாத ஒரு வாரத்தைக் குறிப்பிடுகிறது. பங்கேற்பாளரை ஒரு சராசரியான காலத்திற்கு தெரிவிக்கச் சொல்ல வேண்டாம், | | | Number of days  நாட்களின் எண்ணிக்கை  Don't Know: 77  தெரியாது | | |  |  | | --- | --- | | *If zero days go to D3*  நாட்களின் எண்ணிக்கை 00 எனில் D3 செல்லவும் | | | |
| D2 | How many **servings** of fruits do you eat on **one** of those days?  அத்தகைய நாட்களில், ஒரு நாளில் நீங்கள் எத்தனை குறிப்பிட்ட அளவுகள் பழங்கள் சாப்பிடுகிறீர்கள்?  *(Use showcard)*  *(கார்டை உபயோகிக்கவும்)*  Ask the participant to think of one day he/she can recall easily. Refer to the showcard for serving sizes. Assist them in estimating right serving size.  பங்கேற்பாளரை அவர் நினைவிற்கு ஒரு நாளை நினைத்துப் பார்க்கச் சொல்லவும் . பரிமாறப்படும் அளவுகளுக்கான கார்டைப் பார்க்கவும். பரிமாறப்படும் சரியான அளவை மதிப்பீடு செய்வதில் அவர்களுக்கு உதவவும் | | | Number of servings  பரிமாறப்படும் முறைகளின் எண்ணிக்கை  Don't Know: 77  தெரியாது | | |  |  | | --- | --- | |  | | | |
| D3 | In a typical week, how many days do you **eat vegetables**?  ஒரு வழக்கமான வாரத்தில், எத்தனை நாட்கள் நீங்கள் **காய்கறிகளைச் சாப்பிடுகிறீர்கள்**?  *(Use showcard)*  *(கார்டை உபயோகிக்கவும்)*  Ask the participant to think of any vegetable on the showcard. A typical week means a "normal" week when the diet is notaffected by cultural, religious, or other events. Ask the participant to not report an average over a period.  பங்கேற்பாளரை கார்டின் மீதுள்ள ஏதாவது காய்கறியை நினைக்கச் சொல்லவும். ஒரு வழக்கமான வாரம் என்பது ஒரு “சாதாரணமான” வாரத்தை குறிப்பிடுகிறது, இதில், கலாச்சாரம், மதம் அல்லது மற்ற நிகழ்ச்சிகளால் உணவு பாதிக்கப்படாத ஒரு வாரத்தைக் குறிப்பிடுகிறது. பங்கேற்பாளரை ஒரு சராசரியான காலத்திற்கு தெரிவிக்கச் சொல்ல வேண்டாம். | | | Number of days  நாட்களின் எண்ணிக்கை  Don't Know: 77  தெரியாது | | |  |  | | --- | --- | | *If zero days go to D5*  நாட்களின் எண்ணிக்கை  00 எனில் D5 செல்லவும் | | | |
| D4 | How many **servings** of vegetables do you eat on one of those days?  அத்தகைய நாட்களில், ஒரு நாளில் நீங்கள் எத்தனை குறிப்பிட்ட அளவுகள் காய்கறிகளைச் சாப்பிடுகிறீர்கள்?  *(Use showcard)*  *(கார்டை உபயோகிக்கவும்)*  Ask the participant to think of one day he/she can recall easily. Refer to the showcard for serving sizes. Assist them in estimating right serving size.  பங்கேற்பாளரை அவர் சுலபமாக நினைவுபடுத்திச் சொல்லக்கூடிய ஒரு நாளை நினைக்கச் சொல்லவும். பரிமாறப்படும் அளவுகளுக்கு கார்டைப் பார்க்கவும். பரிமாறப்படும் அளவை மதிப்பீடு செய்வதற்கு அவர்களுக்கு உதவவும் | | | Number of servings    பரிமாறப்படுவதன் எண்ணிக்கை  Don't Know: 77  தெரியாது | | |  |  | | --- | --- | |  | | | |
| **Dietary Salt உணவில் உப்பு** | | | | | | | | | | |
| With the next questions, we would like to learn more about salt in your diet.  அடுத்த கேள்விகளில், உங்கள் உணவில் உள்ள உப்பு பற்றி நாங்கள் தெரிந்து கொள்ள விரும்புகிறோம்.   - *Use show card; கார்டு உபயோகிக்கவும்*   The following questions pertain to adding salt to the food right before you eat it, food preparation at your home and on controlling your salt intake. Please answer the questions even if you consider yourself taking a diet low in salt.  பின்வரும் கேள்விகள் நீங்கள் சாப்பிடுவதற்கு முன் உணவில் சேர்க்கும் உப்பு, உங்கள் வீட்டில் உணவு தயாரிப்பது மற்றும் நீங்கள் உப்பு சாப்பிடுவதைக் கட்டுப்படுத்துவது தொடர்பானது. நீங்கள் உணவில் உப்பு குறைவாக எடுப்பதாக கருதினாலும் இந்த கேள்விகளுக்கு பதில் அளிக்கவும்.  Read this opening statement out loud. Don't forget to use the showcard which will help the respondent when answering to the questions  இந்த தொடக்க வாக்கியத்தை சப்தமாக வாசிக்கவும். இந்தக் கேள்விகளுக்கு பதில் அளிக்கும் போது கார்டை உபயோகிப்பதற்கு மறக்க வேண்டாம் | | | | | | | | | | |
| D7 | | How often do you add extra salt to your food right before you eat it or as you are eating it? எவ்வப்போது நீங்கள் சாப்பிடும்போது அல்லது சாப்பிடுவதற்கு முன் அதிகமான உப்பை சேர்த்துக் கொள்வீர்களா?  Just take the participant opinion, do not ask leading question  பங்கேற்பாளரின் அபிப்பிராயத்தை பெறவும், வழிகாட்டும் வகையில் கேள்வி கேட்க வேண்டாம் | Never….0  எப்பொழுதும் இல்லை  Always….1  எப்பொழுதும்  Often….2  அடிக்கடி  Sometimes….3  சில நேரங்களில்  Rarely….4  அரிதாக  Don’t know….7  தெரியாது | | | | | |  | | --- | |  | |
| D8 | | How often do you eat food high in salt?  High salt food include salty snacks, pickles, fried food such as pakkoda, kurkure, chips, papad, sauce / Chuttney, processed meat in tin.  எவ்வப்போது நீங்கள் எவ்வளவு உப்பு அதிகம் உள்ள உணவுகளை எடுத்துக் கொள்வீர்கள்.  நொறுக்குத்தீனி, ஊறுகாய், பொறிக்கப்பட்ட உணவு வகைகள், பக்கோடா, குர்குரே, சிப்ஸ், அப்பளம், சாஸ், சட்னி மற்றும் டப்பாவில் அடைத்த பதப்படுத்தப்பட்ட இறைச்சி | Never….0  எப்பொழுதும் இல்லை  Always….1  எப்பொழுதும்  Often….2  அடிக்கடி  Sometimes….3  சில நேரங்களில்  Rarely….4  அரிதாக  Don’t know….7  தெரியாது | | | | | |  | | --- | |  | |
| D9 | | On an average how many meals per week do you eat that were not prepared at home? சராசரியாக ஒரு வாரத்தில் எத்தனை நாட்கள் நீங்கள் வீட்டில் சமைக்காத உணவுகளை (காலை உணவு/ மதிய உணவு/ இரவு உணவு) சாப்பிடுவீர்கள்?  *By meals I mean break fast/ lunch/dinner* |  | | | | | |  | | --- | |  | |
| D10 | | How often do you eat at quick serve locations (fast food restaurants, food cart, stalls)? எவ்வப்போது நீங்கள் எவ்வளவு முறை துரித உணவகம், ரோட்டு கடை, உணவு வண்டி போன்றவற்றில் சாப்பிடுவீர்கள்?  Just take the participant opinion, do not ask leading question  பங்கேற்பாளர் அபிப்பிராயத்தை மட்டும் பெறவும், தூண்டும் கேள்விகள் எதையும் கேட்க வேண்டாம் | Never…..0  எப்பொழுதும் இல்லை  Always…..1  எப்பொழுதும்  Often…..2  அடிக்கடி  Sometimes…..3  சில நேரங்களில்  Rarely…..4  அரிதாக  Don’t know…..7  தெரியாது | | | | | |  | | --- | |  | |
| D11 | | Do you think that consuming too much salt or salty food could causes health problem? உணவில் அதிக உப்பு அல்லது உப்பினால் ஆன உணவுகளை சாப்பிடுவதால் ஆரோக்கியத்தை பாதிக்கிறது என நீங்கள், நினைக்கிறீர்களா?  Just take the participant opinion, do not ask leading question  பங்கேற்பாளர் அபிப்பிராயத்தை மட்டும் பெறவும், தூண்டும் கேள்விகள் எதையும் கேட்க வேண்டாம் | Yes…1  ஆம்  No…2  இல்லை  Don’t know…7  தெரியாது | | | | | |  | | --- | |  | |
| D12 | | How important to you is lowering of salt in your diet?  நீங்கள் உணவில் உப்பை குறைவாக எடுத்துள்கொள்வது எவ்வளவு முக்கியமென நினைக்கிறீர்கள்? | VeryImportant…….1  மிக முக்கியம்  Somewhat Important…..2  ஓரளவு முக்கியமான  Not at all important…..3  முக்கியம் இல்லை  Don’t know……7  தெரியாது | | | | |  | | --- | | | |  |
| D13 | | Do you practice any of the following on a regular basis to control your salt intake?  (Record for each)  நீங்கள் அன்றாடம் உப்பு எடுத்துக்கொள்வதை கட்டுப்படுத்துவதற்கு வழக்கமான அடிப்படையில் பின்வரும் எதையாவது செய்கிறீர்களா (ஒவ்வொன்றுக்கும் பதிவு செய்யவும்)  Select the appropriate response for each option. Ask the participant to only consider actions that he/she undertakes specifically to control salt intake, and not for any other purpose  ஒவ்வொரு வாய்ப்பிற்கும் பொருத்தமான பதிலைத் தேர்ந்தெடுக்கவும். பங்கேற்பாளரை குறிப்பாக உப்பு எடுத்துக் கொள்வதைக் கட்டுப்படுத்துவதற்கு அவர் மேற்கொள்ளும் நடவடிக்கைகளை மட்டும் கருதச் சொல்லவும் | | | | | | | | |
| D13a | | Limit consumption of high salt containing foods like papad, pickles, dry fishes, etc.  நீங்கள் உப்பு அதிகமாக உள்ள அப்பளம், ஊறுகாய், கருவாடு போன்ற உணவுகளை குறைத்துக் கொண்டீர்களா? | | | Yes ....1  ஆம்  No ...2  இல்லை | | |  | | --- | | |  | |
| D13b | | Look at the salt or sodium content on food labels  நீங்கள் உணவு பொருட்கள் வாங்கும் பொழுது அதன் உறையின் மீது உள்ள உப்பு / சோடியம் அளவை பார்ப்பதுண்டா? | | | Yes ....1  ஆம்  No ...2  இல்லை | | |  | | --- | | |  | |
| D13c | | Buy low salt/sodium alternatives (but mention this only if you use alternatives to reduce the sodium content and not if you take these as a habit or for taste).  உப்பு / சோடியம் குறைவாக உள்ள மாற்று உப்பு வகைகள் வாங்குவது (உப்பு / சோடியம் அளவை குறைக்கும் நோக்கத்தில் செய்திருந்தால் மட்டுமே இக்கேள்விக்கான பதிலை பதிவு செய்யவும். இது பழக்கத்திர்காகவோ அல்லது சுவைக்காகவோ செய்திருந்தால் பதிலை பதிவு செய்ய வேண்டிய அவசியமில்லை) | | | Yes ....1  ஆம்  No ...2  இல்லை | | |  | | --- | | |  | |
| D13d | | Add less salt and use other spices to make up for the taste when cooking?  நீங்கள் சமைக்கும் போது உப்பை குறைத்து, சுவைக்காக மற்ற மசாலாக்களை உபயோகிர்பீப்ர்களா? | | | Yes ....1  ஆம்  No ...2  இல்லை  Don’t know……7  தெரியாது | | |  | | --- | | |  | |
| D13e | | Avoid eating foods prepared outside of a home.  வெளியில் தயாரிக்கப்பட்ட உணவுகளை சாப்பிடுவதை தவிர்க்கிறீர்காளா?  Ask the participant to think of meals that were NOT prepared at home, (Home includes his/her own home, the home of other family members or friends)  பங்கேற்பாளரை வீட்டில் தயாரிக்கப்படாத உணவுகளை நினைத்துப் பார்க்கச் சொல்லவும், (வீடு என்பது அவருடைய சொந்த வீடு, மற்ற குடும்ப உறுப்பினர்கள் அல்லது நண்பர்கள் வீட்டை உள்ளடக்கியது) | | | Yes....1  ஆம்  No...2  இல்லை | | |  | | --- | | |  | |
| D13f | | Not adding salt on table or not adding salt in rice/ flour  நீங்கள் உட்கொள்ளும் உப்பு அளவைக் கட்டுப்படுத்துவதற்காக டேபிலில் உப்பை சேர்க்காமல் இருப்பது அல்லது சாதம்/மாவில உப்பை சேர்க்காமல இருப்பது | | | Yes....1  ஆம்  No...2  இல்லை  Don’t know…7  தெரியாது | | |  |  | | --- | --- | | |  | |
| Cooking habits and use of oil சமைக்கும் பழக்கங்கள் மற்றும் எண்னெய் உபயோகம் | | | | | | | | | | |
| D14 | | Which of the following types of fuel do you use for cooking?  நீங்கள் சமைப்பதற்காக பின்வரும் எரிபொருள் வகைகளில் எவற்றைப் பயன்படுத்துகிறீர்கள்?  *(You can also ask the other members of the family if required)*  தேவைப்பட்டால், வீட்டில் உள்ள பிற நபர்களிடமும் கேட்கலாம் | | | | | | | | |
| D14 a | | Electricity  மின்சாரம் | | | Yes .....1  ஆம்  No ...2  இல்லை | | |  | | --- | | |  | |
| D14 b | | LPG/Natural gas  எல்பிஜி/ இயற்கை எரிவாயு | | | Yes .....1  ஆம்  No ...2  இல்லை | | |  | | --- | | |  | |
| D14c | | Biogas  சாண எரிவாயு (பயோ கேஸ்) | | | Yes .....1  ஆம்  No ...2  இல்லை | | |  | | --- | | |  | |
| D14d | | Kerosene  மண்ணெண்ணெய் | | | Yes .....1  ஆம்  No ...2  இல்லை | | |  | | --- | | |  | |
| D14e | | Coal/lignite  நிலக்கரி/ லிக்னைட் | | | Yes .....1  ஆம்  No ...2  இல்லை | | |  | | --- | | |  | |
| D14 f | | Charcoal  கரித்துண்டு | | | Yes .....1  ஆம்  No ...2  இல்லை | | |  | | --- | | |  | |
| D14g | | Wood  விறகு | | | Yes .....1  ஆம்  No ...2  இல்லை | | |  | | --- | | |  | |
| D14h | | Straw/shrubs/grass  வைக்கோல்/காய்ந்த  செடிகொடி/ புல் | | | Yes .....1  ஆம்  No ...2  இல்லை | | |  | | --- | | |  | |
| D14i | | Agricultural crop waste (corn, sesame, bajra, jowar…)  விவசாயக் கழிவு (சோளம், எள், கம்பு, மக்க சோளம்) | | | Yes .....1  ஆம்  No ...2  இல்லை | | |  | | --- | | |  | |
| D14j | | Dung cakes  சாண வரட்டி | | | Yes .....1  ஆம்  No ...2  இல்லை | | |  | | --- | | |  | |
| D14k | | Others(specify)  மற்றவை (குறிப்பிடவும்) | | | Yes .....1  ஆம்  No ...2  இல்லை | | |  | | --- | | |  | |
| D15 | | Among those listed above what type of fuel does your household mainly use for cooking?  மேலே பட்டியிலிடப் பட்டுள்ளவற்றிடையே  சமைப்பதற்காக முதன்மையாக உங்களது வீட்டில் எதைப் பயன்படுத்துகிறீர்கள்?  *(You can also ask the other members of the family if required)*  தேவைப்பட்டால், வீட்டில் உள்ள பிற நபர்களிடமும் கேட்கலாம் | | | Electricity……1  LPG/Natural gas……2  Biogas….3  Kerosene….4  Coal/lignite….5  Charcoal….6  Wood….10  Straw/shrubs/grass….11  Agricultural crop waste  ……… 12  Dung cakes……. 13  Others(specify)…. 14  Do not know----7 | | |  | | --- | | |  | |
| D16 | | What are the types of oil or fat used for meal preparation in your household?  உங்களது குடும்பத்தில் சாப்பாடு தயாரிப்பதற்காக என்ன வகை எண்ணெய் அல்லது  கொழுப்பு பயன்படுத்தப்படுகின்றன?  *(You can also ask the other members of the family if required)*  தேவைப்பட்டால், வீட்டில் உள்ள பிற நபர்களிடமும் கேட்கலாம் | | | | | | | | |
| D16a | | Oil use  எண்ணெய் பயன்பாடு | | | Use some oil….1  Don’t use any oil/don’t know….2 | | |  | | --- | | | *If Don’t use any oil/don’t know go to P1* | |
| D16b | | Mustard Oil  கடுகு எண்ணெய் | | | Yes .....1  ஆம்  No ...2  இல்லை | | |  | | --- | | |  | |
| D16c | | Coconut oil  தேங்காய் எண்ணெய் | | | Yes .....1  ஆம்  No ...2  இல்லை | | |  | | --- | | |  | |
| D16d | | Groundnut oil  கடலை எண்ணெய் | | | Yes .....1  ஆம்  No ...2  இல்லை | | |  | | --- | | |  | |
| D16e | | Sunflower oil  சூரிய காந்தி எண்ணெய் | | | Yes .....1  ஆம்  No ...2  இல்லை | | |  | | --- | | |  | |
| D16f | | Soyabean oil  சோயபீன் எண்ணெய் | | | Yes .....1  ஆம்  No ...2  இல்லை | | |  | | --- | | |  | |
| D16g | | Palm oil  பாமாயில் | | | Yes .....1  ஆம்  No ...2  இல்லை | | |  | | --- | | |  | |
| D16h | | Vanaspati  வனஸ்பதி | | | Yes .....1  ஆம்  No ...2  இல்லை | | |  | | --- | | |  | |
| D16i | | Pure Ghee  சுத்தமான நெய் | | | Yes .....1  ஆம்  No ...2  இல்லை | | |  | | --- | | |  | |
| D16j | | Butter  வெண்ணெய் | | | Yes .....1  ஆம்  No ...2  இல்லை | | |  | | --- | | |  | |
| D16k | | Sesame oil  நல்லெண்ணெய் | | | Yes .....1  ஆம்  No ...2  இல்லை | | |  | | --- | | |  | |
| D16l | | Others(specify) | | | Yes .....1  ஆம்  -----------------  No ...2  இல்லை | | |  | | --- | | |  | |
| D17 | | What type of oil or fat is the most often used for meal preparation in your household?  *(select only one)*  உங்கள் வீட்டில் சமையலுக்கு எந்த வகை எண்ணெய்  அல்லது கொழுப்பு அதிகம் பயன்படுத்தப்படுகிறது?  *(You can also ask the other members of the family if required)*  தேவைப்பட்டால், வீட்டில் உள்ள பிற நபர்களிடமும் கேட்கலாம் | | | Mustard Oil….1  Coconut oil…2  Groundnut oil…3  Sunflower oil…4  Soyabean oil….5  Palm oil…6  Vanaspati…10  Pure Ghee….11  Butter….12  Sesame oil….13  Can’t decide/None in particular….14  Others(specify)….15  -----------------  Rice bran oil….16  Don’t know….7 | |  | |  | |

| Physical Activity | | | | | | |
| --- | --- | --- | --- | --- | --- | --- |
| I am going to ask you about the time you spend doing different types of physical activity in a typical week. Please answer these questions even if you do not consider yourself to be a physically active person.  In answering the following questions,  ‘Moderate-Intensity activities’ are activities that require effort and cause small increases in breathing or heart rate.  ‘Vigorous –Intensity activities’ are activities that require hard physical effort and cause large increase in breathing or heart rate.  The respondent will have to think first about the time he/she spends doing work (paid or unpaid work, household chores, harvesting food, fishing or hunting for food, seeking employment [Insert other examples if needed]) and about the time spent in vigorous as well as moderate physical activity during leisure time.  பேட்டியளிப்பவர் முதலில் அவர் வேலை செய்வதில் செலவழிக்கும் நேரம் பற்றி மற்றும் ஓய்வு நேரத்தின் போது மேற்கொள்ளும் தீவிரமான மற்றும் மிதமான அளவு உடல் சார்ந்த நடவடிக்கைகளில் செலவழிக்கும் நேரம் பற்றி நினைத்துப் பார்க்க வேண்டும்.  பேட்டியளிப்பவர் பின்வரும் கேள்விக்கு பதில் அளிக்கும் போது,  ‘மிதமான-அளவு நடவடிக்கைகள்’ என்பது மிதமான உடல் உழைப்பு சார்ந்த முயற்சி தேவைப்படுவது மற்றும் குறைவாக சுவாசிப்பதை அல்லது இதய துடிப்பை அதிகரிக்கிறது என்பதை அவருக்கு நினைவுபடுத்தவும்  ‘தீவிர-அளவிலான நடவடிக்கைகள்’ என்பது கடினமான உடல் உழைப்பு தேவைப்படுவது மற்றும் அதிக அளவில் சுவாசம் அல்லது இருதய துடிப்பை அதிகரிக்கறிது, என்பதை அவருக்கு நினைவுபடுத்தவும்  பேட்டியளிப்பவர் பதில் அளிக்கும் போது கார்டை உபயோகிப்பதற்கு மறக்க வேண்டாம் , இது அவருக்கு உதவும் | | | | | | |
| **Questions** **கேள்விகள்** | | **Response****பதில்கள்** |  | | **Skip** |  |
| P1 | Does your work at home/work place involve **moderate-intensity** activity that causes small increases in breathing or heart rate, for at least 10 minutes continuously such as brisk walking or carrying loads (<20 kgs), manual washing of clothes, dry sweeping of floor, wet mopping of floor, drawing water from well, carrying water from tap, carrying water from river or well, manual grinding or pounding of cereals, gardening at home etc. ?  உங்கள் வேலை என்பது சுறுசுறுப்பாக நடப்பது அல்லது லோடுகளை ஏற்றிச் செல்வது (<20 kgs), கைகளால் துணிகளைத் துவைப்பது, தரையைப் பெருக்குவது, துடைப்பது, கிணற்றிலிருந்து தண்ணீர் எடுப்பது, குழாயிலிருந்து தண்ணீர் எடுத்துச் செல்வது, ஆறு அல்லது கிணற்றிலிருந்து தண்ணீர் எடுத்துச் செல்வது, தானியங்களை கைகளால் இயக்கும் சாதனங்களுடன் அரைப்பது அல்லது இடிப்பது, வீட்டில் தோட்ட வேலை போன்ற குறைந்தபட்சம் 10 நிமிடங்களுக்கு தொடர்ச்சியாக சுவாசிப்பதை அல்லது இருதய துடிப்பை சிறிய அளவில் அதிகரிக்கும் மிதமான-அளவு நடவடிக்கையை உள்ளடக்கியிருக்கிறதா?  *(Use show card)*  *(கார்டை உபயோகிக்கவும்)* | Yes ....1  ஆம்  No ...2  இல்லை  Not applicable…9 பொருந்தாது | |  | | --- | | | *If No, go toP4*  *இல்லை என்றால். P4க்கு செல்லவும்* |  |
| P2 | In a typical week, on how many days do you do **moderate-intensity activities** as part of your work at home/ workplace for atleast 10minutes continuously.  ஒரு வழக்கமான வாரத்தில், எத்தனை நாட்கள் தொடர்ச்சியாக குறைந்தபட்சம் 10 நிமிடங்களுக்கு உங்கள் வேலையின் ஒரு பகுதியாக நீங்கள் மிதமான-அளவு நடவடிக்கைகளை மேற்கொள்கிறீர்கள் ?  Enter '77' if not known.  “Typical week” means a week when the participant is engaged in his/her usual activities. Valid responses range from 1-7.  ”வழக்கமான வாரம்” என்பது பங்கேற்பாளர் அவருடைய வழக்கமான நடவடிக்கைகளில் ஈடுபட்டிருக்கும் வாரத்தைக் குறிப்பிடுகிறது. | Number of days  நாட்களின் எண்ணிக்கை | |  | | --- | | |  |  |
| P3 | On a typical day how much time do you spend doing **moderate-intensity** activity at home/work place?  ஒரு வழக்கமான நாளில், வீட்டில் / வேலை செய்யும் இடத்தில் **மிதமான-அளவிலான** நடவடிக்கைகளைச் செய்வதில் நீங்கள் எவ்வளவு நேரம் செலவழிக்கிறீர்கள் ?  Enter values in both fields.  Enter '77' in both fields if not known  Ask the participant to think of a typical day he/she can recall easily in which he/she engaged in moderate-intensity activities at work. The participant should only consider those activities undertaken continuously for 10 minutes or more. Probe very high responses (over 4 hrs) to verify.  பங்கேற்பாளரை, வேலையில் மிதமான-அளவிலான நடவடிக்கைகளில் அவர் ஈடுபட்டிருந்த, சுலபமாக நினைவிற்கு வரும் ஒரு வழக்கமான நாளை நினைத்துப் பார்க்கச் சொல்லவும், தொடர்சியாக 10 நிமிடங்கள் அல்லது அதிகமாக மேற்கொள்ளப்படும் நடவடிக்கைகளை மட்டுமே பங்கேற்பாளர் கருத வேண்டும். 4 மணி நேரங்களுக்கும் அதிகமாக என்றால் பதில்களை சரிபார்ப்பதற்கு ஆராயவும் | Hours  மணி நேரங்கள்  Minutes  நிமிடங்கள் | |  |  | | --- | --- | |  |  | | |  |  |
| P4 | Does your routine work at home/workplace involve vigorous-intensity activity that causes large increases in breathing or heart rate, for atleast 10 minutes continuosly such as carrying or lifting heavy loads, digging or construction work?  உங்கள் வழக்கமான வேலை. தொடர்சியாக குறைந்தபட்சம் 10 நிமிடங்களுக்கு சுவாசிப்பதை அல்லது இதயத் துடிப்பை அதிக அளவில் அதிகரிக்கும் தீவிர-அளவிலான நடவடிக்கையை உள்ளடக்கியிருக்கிறதா? (கனமான பொருட்களை எடுத்துச் செல்வது அல்லது தூக்குவது, குழி வெட்டுவது அல்லது கட்டுமான வேலை போன்றவை) ?  *(Use show card)*  *(கார்டை உபயோகிக்கவும்)* | Yes .....1  ஆம்  No ...2  இல்லை  Not applicable …9  பொருந்தாது | |  | | --- | | | *If No, Go to P7*  *இல்லை என்றால். P7க்கு செல்லவும்*; |  |
| P5 | In a typical week, on how many days do you do vigorous-intensity activities as part of your work at home/ workplace?  ஒரு வழக்கமான வாரத்தில், உங்கள் வேலையின் ஒரு பகுதியாக எத்தனை நாட்கள் நீங்கள் **தீவிர** - அளவிலான நடவடிக்கைகளை வீட்டில் / வேலை செய்யும் இடத்தில் செய்கிறீர்கள் ?  Enter '77' if not known | Number of days  நாட்களின் எண்ணிக்கை | |  | | --- | | |  |  |
| P6 | On a typical day, how much time do you spend doing vigorous-intensity activities at home /work place?  ஒரு வழக்கமான நாளில் வீட்டில் / வேலை செய்யும் இடத்தில் தீவிர**-அளவிலான** நடவடிக்கைகளைச் செய்வதில் நீங்கள் எவ்வளவு நேரம் செலவழிக்கிறீர்கள் ?  Enter values in both fields.  Enter '77' in both fields if not known  Ask the participant to think of a typical day he/she can recall easily in which he/she engaged in vigorous-intensity activities at work. The participant should only consider those activities undertaken continuously for 10 minutes or more. பங்கேற்பாளர் தீவிர -அளவிலான நடவடிக்கைகளில் ஈடுபட்டிருந்தவற்றை சுலபமாக நினைவிற்கு கொண்டு வரும் ஒரு வழக்கமான நாளை நினைத்துப் பார்க்கச் சொல்லவும். பங்கேற்பாளர் 10 நிமிடங்கள் அல்லது அதிகமாக தொடர்சியாக மேற்கொண்டிருக்கும் நடவடிக்கைகளை மட்டும் கருதவும். 4 மணி நேரங்களுக்கும் அதிகமாக என்றால் பதில்களை சரிபார்ப்பதற்கு ஆராயவும். | Hoursமணிநேரங்கள்Minutesநிமிடங்கள் | |  |  | | --- | --- | |  |  | | |  |  |
| While answering the next question please exclude any activities that you have already mentioned in the questions on intensive or moderate physical activity. Now I would like to ask you about sports, fitness and recreational activities  அடுத்த கேள்விக்கு பதிலளிக்கும் போது முன்பு குறிப்பிட்ட பதில்களை தவிர்க்கவும். நான் தற்போது விளையாட்டு, உடற்பயிற்சி மற்றும் பொழுதுபோக்குப் பற்றி கேட்கின்றேன். | | | | | |  |
| P7 | Do you do any moderate-intensity sports, fitness or recreational (leisure) activities that cause small increases in breathing or heart rate such as brisk walking, cycling, gym fitness exercise, yoga etc., for at least 10 minutes continuously?  தொடர்ச்சியாக 10 நிமிடம் உங்கள் சுவாசத்தை அல்லது இதயத் துடிப்பை அதிகரிக்கும் மிதமான-அளவிலான விளையாட்டு, உடற்பயிற்சி அல்லது-பொழுதுபோக்கு, போன்றவற்றை செய்கிறீர்களா? (மிதிவண்டி ஓட்டுவது, நடைப்பயிற்சி, யோகா)  *(Use show card)*  *(கார்டை உபயோகிக்கவும்)* | Yes....1  ஆம்  No ...2  இல்லை  Not applicable…9  பொருந்தாது | |  | | --- | | | *If No, go to P10*  *இல்லை என்றால். P10க்கு செல்லவும்*; |  |
| P8 | In a typical week, on how many days do you do moderate-intensity sports, fitness or recreational (leisure) activities?  ஒரு வழக்கமான வாரத்தில், எத்தனை நாட்களில் நீங்கள் மிதமான-அளவிலான விளையாட்டு , உடல் உறுதி அல்லது பொழுதுபோக்கு நடவடிக்கைளை செய்கிறீர்கள் ? | Number of days  நாட்களின் எண்ணிக்கை | |  | | --- | | |  |  |
| P9 | On a typical day, how much time do you spend doing moderate-intensity sports, fitness or recreational activities?  ஒரு வழக்கமான நாளில், நீங்கள் ஏதாவது மிதமான-அளவிலான விளையாட்டுகள் , உடல் உறுதிக்கான அல்லது பொழுதுபோக்கிற்கான நடவடிக்கைகையை மேற்கொள்ளும் பொழுது நீங்கள் எவ்வளவு நேரம் செலவழிக்கிறீர்கள் ?  Enter values in both fields.  Enter '77' in both fields if not known. | Hoursமணிநேரங்கள்Minutesநிமிடங்கள் | |  |  | | --- | --- | |  |  | | |  |  |
| P10 | Do you do any **vigorous**-intensity **sports**, fitness or recreational (leisure) activities that cause large increases in breathing or heart rate like (running or football) for at **least 10 minutes continuously**?  தொடர்ச்சியாக குறைந்தபட்சம் 10 நிமிடங்களுக்கு சுவாசிப்பதை அல்லது இருதய துடிப்பை அதிகமாக அதிகரிக்கும் (ஓடுவது அல்லது கால்பந்து) ஏதாவது தீவிர-அளவிலான விளையாட்டுகள், உடல் உறுதிக்கான அல்லது பொழுது போக்கு நடவடிக்கைகள் எதையாவது நீங்கள் செய்கிறீர்களா ?  Ask the participant to think about recreational vigorous-intensity activities only. Activities are regarded as vigorous intensity if they cause large increases in breathing and/or heart rate  *பங்கேற்பாளரை தீவிர-அளவிலான பொழுதுபோக்கு நடவடிக்கைகளைப் பற்றி மட்டுமே நினைத்துப் பார்க்கச் சொல்லவும். சுவாசிப்பதில் மற்றும் / அல்லது இருதய துடிப்பை அதிகளவில் அதிகரிப்பதற்கு காரணமாக இருந்தால் அந்த நடவடிக்கைகள் தீவிர-அளவிலான நடவடிக்கைகளாக கருதப்படும்.* 4 மணி நேரங்களுக்கும் அதிகமாக என்றால் பதில்களை சரிபார்ப்பதற்கு ஆராயவும். | Yes....1  ஆம்  No ...2  இல்லை  Not applicable....9  பொருந்தாது | |  | | --- | | | *If No, go to P13*  *இல்லை என்றால். P13க்கு செல்லவும்* |  |
| P11 | In a typical week, on how many days do you do vigorous-intensity sports, fitness, or recreational activity?  ஒரு வழக்கமான வாரத்தில், எத்தனை நாட்களில் நீங்கள் தீவிர-அளவிலான விளையாட்டு, உடல் உறுதி அல்லது பொழுதுபோக்கு நடவடிக்கைளை செய்கிறீர்கள் ? | Number of days  நாட்களின் எண்ணிக்கை | |  | | --- | | |  |  |
| P12 | On a typical day, how much time do you spend doing vigorous-intensity sports, fitness or recreational (leisure) activities?  ஒரு வழக்கமான நாளில், நீங்கள் ஏதாவது தீவிர-அளவிலான விளையாட்டுகள், உடல் உறுதிக்கான அல்லது பொழுதுபோக்கிற்கான நடவடிக்கைகையை மேற்கொள்ள எவ்வளவு நேரம் செலவழிக்கிறீர்கள் ?  Ask the participant to think of a typical day he/she can recall easily in which he/she engaged in recreational vigorous-intensity activities. The participant should only consider those activities undertaken continuously for 10 minutes or more. Probe very high responses (over 4 hrs) to verify.  Enter values in both fields.  Enter '77' in both fields if not known.  பங்கேற்பாளர் தீவிர-அளவிலான விளையாட்டுகள், உடல் உறுதிக்கான அல்லது பொழுதுபோக்கிற்கான நடவடிக்கைககளில் ஈடுபட்டிருந்தவற்றை சுலபமாக நினைவிற்கு கொண்டு வரும் ஒரு வழக்கமான நாளை நினைத்துப் பார்க்கச் சொல்லவும். பங்கேற்பாளர் 10 நிமிடங்கள் அல்லது அதிகமாக தொடர்சியாக மேற்கொண்டிருக்கும் நடவடிக்கைகளை மட்டும் கருதவும். 4 மணி நேரங்களுக்கும் அதிகமாக என்றால் பதில்களை சரிபார்ப்பதற்கு ஆராயவும். | Hoursமணிநேரங்கள்Minutesநிமிடங்கள் | |  |  | | --- | --- | |  |  | | |  |  |
| P13 | During a typical or usual day, how much time do you spend sitting or reclining and watching television, working on computer, playing game in mobile/tablet, talking with friends, or doing other sitting activities like knitting, embroidery etc.? Please also include the time spent in sitting in office. But DO NOT include time spent sleeping.  ஒரு வழக்கமான நாளில், உட்காருவதில் அல்லது சாய்ந்து டிவி பார்பதில், கம்ப்யூட்டரில் வேலை செய்வதில், மொபைல் / டேப்லெட்டில் கேம் விளையாடுவது, நண்பர்களுடன் பேசுவது அல்லது பின்னுவது, எம்ப்ராய்டரி போன்ற மற்ற உட்கார்ந்து செய்யும் நடவடிக்கைகளில் நீங்கள் எவ்வளவு நேரம் செலவழிக்கிறீர்கள்? அலுவலகத்தில் உட்கார்ந்து செலவழிக்கும் நேரத்தையும் சேர்த்துக் கொள்ளவும். ஆனால் தூங்குவதற்கு செலவழித்த நேரத்தைச் சேர்க்க வேண்டாம். | Hoursமணிநேரங்கள்Minutesநிமிடங்கள் | |  |  | | --- | --- | |  |  | | |  |  |
| P14 | Do you practice **Yoga (which includes activities like asana, pranayam or mediation)**?  நீங்கள் யோகா பயிற்சி செய்கிறீர்களா ? (ஆசனம்/ பிரணாயாமம் அல்லது தியானம் சேர்ந்தது) | Yes .....1  ஆம்  No ...2  இல்லை | |  | | --- | | | If No, go to H1 |  |
|  | If yes, how many days in a typical week do you perform the following (complete one row)  ஆம் என்றால், ஒரு வழக்கமான வாரத்தில் எத்தனை நாட்கள் நீங்கள் பின்வருவதைச் செய்கிறீர்கள்(ஒரு வரிசையைப் பூர்த்தி செய்யவும்)  Enter 77 if Not known  Enter 99 if Not doing  தெரியாவிட்டால், 77 எழுதவும்  செய்யாவிட்டால், 99 எழுதவும் | Number of days in the week  வாரத்தில் நாட்களின் எண்ணிக்கை | Number of sittings in a typical day that you perform these  ஒரு வழக்கமான நாளில் இவற்றை நீங்கள் எத்தனை முறை செய்கிறீர்கள் | |  |  |
|  |  |  | Hours மணி நேரங்கள் | Minutes நிமிடங்கள் |  |  |
| P14a | Asana  ஆசனம் | |  | | --- | | |  |  | | --- | --- | | |  |  | | --- | --- | |  |  |
| P14b | Pranayam  பிரணாயாமம் | |  | | --- | | |  |  | | --- | --- | | |  |  | | --- | --- | |  |  |
| P14c | Meditation  தியானம் | |  | | --- | | |  |  | | --- | --- | | |  |  | | --- | --- | |  |  |

| **History of Raised Blood Pressure** **உயர் இரத்த அழுத்த வரலாறு** | | | | | | | | | | | | | | | | |
| --- | --- | --- | --- | --- | --- | --- | --- | --- | --- | --- | --- | --- | --- | --- | --- | --- |
| Now I shall ask you about raised blood pressure.இப்போது நான் உங்களிடம் உயர் இரத்த அழுத்தம் பற்றி கேட்க போகிறேன். | | | | | | | | | | | | | | | | |
| **Questions கேள்விகள்** | | | | | **Response பதில்** | | | | | **Skip** | | | | | | |
| H1 | Have you **ever** had your blood pressure measured by a doctor or other health worker?  உங்கள் இரத்த அழுத்தத்தை எப்போதாவது மருத்துவரிடமோ அல்லது சுகாதார ஊழியரிடமோ அளவிட்டிருக்கிறீர்களா?  Ask the participant to only consider measurements done by a doctor or other health worker  பங்கேற்பாளரை ஒரு மருத்துவர் அல்லது மற்ற மருத்துவ ஊழியர் மேற்கொண்ட அளவீடுகளை மட்டுமே கருதச் சொல்லவும் | | | | Yes…1  ஆம்  No …2  இல்லை | | | |  | | --- | | | *If No, go to H11*  *இல்லை என்றால்.H11க்கு செல்லவும்* | | | | | | |
| H2 | When was your blood pressure last measured by a health professional / worker?  மருத்துவ வல்லுனர் / ஊழியர்கள் கடைசியாக உங்கள் இரத்த அழுத்தத்தை அளவிட்டது எப்போது?  Ask him/her to remember and select the response  அவரை யோசித்துப் பார்த்து பதிலைத் தேர்ந்தெடுக்கச் சொல்லவும் | | | | Within past 6 months  கடந்த 6 மாதங்களுக்குள்……...1  Within past 6-12 months  கடந்த 6-12 மாதங்களுக்குள்.….2  Within past 1-2years  கடந்த 1-2 வருடங்களுக்குள் ……….3  Not within past 2 years  கடந்த 2 வருடத்திற்குள் இல்லை……4 | | | |  | | --- | | | *If not in last 12 months (option 3 or 4 selected), go to H3*  கடந்த 12 மாதங்களுக்குள் (3அல்லது 4 தேர்வானால்) H3க்கு செல்லவும்;;; | | | | | | |
| H2a | If measured in last 12 months where was it done?  கடந்த 12 மாதங்களில் அளவிடப்பட்டது என்றால் எங்கு அளவிடப்பட்டது? | | | | Private doctor’s clinic….1  தனியார் கிளினிக்  Primary Health center / Community Health Center……2  ஆரம்ப அல்லது சமுதாய சுகாதார மையம்  Public/Government (district/taluk) hospital…3  தாலுகா/மாவட்ட மருத்துவமனை  Private hospital….4  தனியார் மருத்துவமனை  Govt. Medical college/state referral Hospital….5  அரசு மருத்துவ கல்லூரி, மருத்துவமனை  Government AYUSH clinic……6  அரசு ஆயுஷ் கிளினிக்  Private AYUSH clinic….10  தனியார் ஆயுஷ் கிளினிக்  Traditional healer….11  பாரம்பரிய வைத்தியர்  RMP….12  பதிவு செய்யப்பட்ட மருத்துவ பயிற்சியாளர்  Pharmacy….13  மருந்தகம்  Sub centre….14  துணை சுகாதார மையம்  Government Screening camp….15  அரசு பரிசோதனை முகாம்  Private / NGO Screening camp….16  தனியார் / தன்னார்வ தொண்டு நிறுவனம் பரிசோதனை முகாம்  None of the facility visited….17  மேற்கண்ட எதுவும் இல்லை  Don’t know….77  தெரியாது  Refused/ No response ….88 விருப்பமில்லை | | | |  | | --- | | |  | | | | | | |
| H3 | Have you ever been told by a doctor or other health worker that you have hypertension?  மருத்துவர் அல்லது மருத்துவ ஊழியர் உங்களுக்கு உயர் இரத்த அழுத்தம் இருப்பதாக எப்போதாவது சொல்லியிருக்கிறார்களா?  Select the appropriate response.  பொருத்தமான பதிலைப் பதிவு செய்யவும் | | | | Yes…1  ஆம்  No …2  இல்லை | | | |  | | --- | | | *If No, go to*  *H11இல்லை என்றால்.H11க்கு செல்லவும்* | | | | | | |
| H4 | During the past 12 months, have you visited a doctor or other health worker for hypertension? நீங்கள் கடந்த 12 மாதங்களில் உங்கள் உயர் இரத்த அழுத்தத்திற்காக மருத்துவர் அல்லது மருத்துவ ஊழியரை பார்த்தீர்களா? | | | | Yes…1  ஆம்  No …2  இல்லை | | | |  | | --- | | | *If No, go to*  *H11இல்லை என்றால்.H11க்கு செல்லவும்* | | | | | | |
| H4a | In the past 12 months, which health facility/hospital did you visit most frequently for hypertension?  நீங்கள் கடந்த 12 மாதத்தில் உயர் இரத்த அழுத்தத்திற்காக பெரும்பாலும் எந்த மருத்துவ வசதி அல்லது மருத்துவமனையை அணுகினீர்கள்? | | | | Private doctor’s clinic….1  தனியார் கிளினிக்  Primary Health center / Community Health Center……2  ஆரம்ப அல்லது சமுதாய சுகாதார மையம்  Public/Governement (district/taluk) hospital…3  தாலுகா/மாவட்ட மருத்துவமனை  Private hospital….4  தனியார் மருத்துவமனை  Govt. Medical college/state referral Hospital….5  அரசு மருத்துவ கல்லூரி, மருத்துவமனை  Government AYUSH clinic……6  அரசு ஆயுஷ் கிளினிக்  Private AYUSH clinic….10  தனியார் ஆயுஷ் கிளினிக்  Traditional healer….11  பாரம்பரிய வைத்தியர்  RMP….12  பதிவு செய்யப்பட்ட மருத்துவ பயிற்சியாளர்  Pharmacy….13  மருந்தகம்  Sub centre….14  துணை சுகாதார மையம்  None of the facility visited….15  மேற்கண்ட எதுவும் இல்லை திரையிடல் முகாம்  Don’t know….77  தெரியாது  Refused/ No response ….88 விருப்பமில்லை | | | |  |  | | --- | --- | | | | | | | If 2,3,5,14  go to H5 | | |
| H5 | If taken treatment in Government facility such as PHC/CHC/Sub-centre/District/sub district hospital/Govt. medical colleges/state referral hospitals, were the tablets available in the facility itself?  நீங்கள் அரசு மருத்துவ நிலையங்களில் சிகிச்சை பெற்றிருந்ததால் பரிந்துரைக்கப்பட்ட அனைத்து மருந்துகளும் அங்கேயே கிடைத்தனவா?  (ஆரம்ப அல்லது சமுதாய சுகாதார மையம், துணை மத்திய, மாவட்ட மருத்துவமனை அரசு மருத்துவ கல்லூரி, மருத்துவமனை) | | | | Always…..1  எப்பொழுதும்  Sometimes….2  சில நேரங்களில்  Never….3  எப்பொழுதும் இல்லை  Don’t know….8  தெரியாது | | | |  | | --- | | | | | |  | |  | |
| H6 | On your most recent visit for hypertension, what health facility did you visit?  உங்கள் உயர் இரத்த அழுத்தத்திற்காக எந்த மருத்துவ வசதியை அண்மையில் அணுகியுள்ளீர்கள்?  Select the appropriate response for each of the following  பின்வரும் ஒவ்வொன்றுக்கும் பொருத்தமான பதிலைத் தேர்ந்தெடுக்கவும் | | | | Private doctor’s clinic….1  தனியார் கிளினிக்  Primary Health center / Community Health Center……2  ஆரம்ப அல்லது சமுதாய சுகாதார மையம்  Public/Governement (district/taluk) hospital…3  தாலுகா/மாவட்ட மருத்துவமனை  Private hospital….4  தனியார் மருத்துவமனை  Govt. Medical college/state referral Hospital….5  அரசு மருத்துவ கல்லூரி, மருத்துவமனை  Government AYUSH clinic……6  அரசு ஆயுஷ் கிளினிக்  Private AYUSH clinic….10  தனியார் ஆயுஷ் கிளினிக்  Traditional healer….11  பாரம்பரிய வைத்தியர்  RMP….12  பதிவு செய்யப்பட்ட மருத்துவ பயிற்சியாளர்  Pharmacy….13  மருந்தகம்  Sub centre….14  துணை சுகாதார மையம்  None of the facility visited….15  மேற்கண்ட எதுவும் இல்லை  Don’t know….77  தெரியாது  Refused/ No response ….88 விருப்பமில்லை | | | | |  | | --- | | | | |  | | |  |
| H7 | For how long were the drugs (medication) issued for hypertension during the most recent visit?  அண்மையில் உங்கள் உயர் இரத்த அழுத்தத்திற்காக எத்தனை நாட்களுக்கு மருந்துகள் வழங்கப்பட்டது? | | | | Less than one week….1  ஒரு வாரத்திற்கும் குறைவான  1 week….2  1 வாரம்  2 weeks….3  2 வாரங்கள்  3 weeks….4  3 வாரங்கள்  1 month….5  1 மாதம் | | |  | | --- | |  | | | | |  | | | |
| H8 | Did you have to pay for any drugs (medication) for hypertension that you received or was prescribed during your most recent visit?  அண்மையில், உங்கள் உயர் இரத்த அழுத்தத்திற்காக மருத்துவர் பரிந்துரைத்த மருந்துகளை வெளியில் பணம் கொடுத்து வாங்கினீர்களா? | | | | Yes……1  ஆம்  No……2  இல்லை | | |  | | --- | |  | | | | |  | | | |
| H9 | In the past two weeks, have you taken any drugs (medication) for hypertension prescribed by a doctor or other health worker?  கடந்த 2 வாரங்களில், நீங்கள் உயர் இரத்த அழுத்தத்திற்காக மருத்துவர் அல்லது மருத்துவ ஊழியரால் பரிந்துரைக்கப்பட்ட மருந்துகளை உட்கொண்டீர்களா? | | | | Yes…..1  ஆம்  No…..2  இல்லை | | |  | | --- | |  | | | | | If yes then go to H11 | | | |
| H10 | What is the reason for not taking medication for hypertension prescribed by a doctor or other health worker?  எந்த காரணத்திற்காக நீங்கள் மருத்துவர் அல்லது மருத்துவ ஊழியரால் பரிந்துரைத்த மருந்துகளை எடுத்துக் கொள்ளவில்லை?  (Multiple options possible)  (ஒன்றுக்கு மேற்பட்ட பதில்கள் சாத்தியம்) | Not prescribed…….1  பரிந்துரைக்கப்படவில்லை  Too expensive/Could not afford….2  மிகவும் விலையுயர்ந்த/வாங்க முடியவில்லை  I didn’t feel sick….3  உடல் நலம் நன்றாக உள்ளது  Side effects….4  பக்க விளைவுகள்  Drugs weren’t working….5  மருந்துகள் வேலை செய்யவில்லை  No health provider /pharmacy nearby….6  மருத்துவர் அல்லது மருத்துவ ஊழியர் / மருந்தகம் அருகில் இல்லை  No one to help me to reach health facility…10  மருத்துவ வசதியை அடைய எனக்கு உதவ யாரும் இல்லை  Too long a wait in health facility….11  மருத்துவமனையில் நீண்ட நேரம் காத்திருத்தல்  Too sick to make the trip….12  உடல்நலக் குறைவால் மருத்துவமனைக்கு செல்ல முடியவில்லை  Do not trust medical care….13  மருத்துவ சேவையை நம்பவில்லை  Do not know where to go….14  எங்கு செல்ல வேண்டும் என்று தெரியவில்லை  Others (Specify) ….15  மற்றவை (குறிப்பிடுக)  Refused/ No response ….88  பதிலளிக்க விருப்பமில்லை | | | | | | |  | | --- | | | | | | | | |  |
| **History of Diabetes**  **சர்க்கரை வியாதி** | | | | | | | | | | | | | | | | |
| H11 | Have you **ever** had your blood sugar been measured by a doctor or health worker?  மருத்துவர் அல்லது மருத்துவ ஊழியர்கள் மூலமாக நீங்கள் எப்போதாவது உங்கள் இரத்த சர்க்கரை அளவை அளவிட்டிருக்கிறீர்களா?  Ask the participant to only consider measurements done by a doctor or other health worker.  பங்கேற்பாளரை ஒரு மருத்துவர் அல்லது மற்ற மருத்துவர் ஊழியர் மேற்கொண்டிருக்கும் அளவீடுகளை மட்டும் கருதச் சொல்லவும் | | | | Yes…1  ஆம்  No...2  இல்லை | | | |  | | --- | | | *If No, go to H21 இல்லை என்றால்.H21க்கு செல்லவும்* | | | | | | |
| H12 | When was your blood sugar last measured by health professional/workers?  மருத்துவ வல்லுநர்கள் / ஊழியர்கள் கடைசியாக எப்போது உங்கள் இரத்த சர்க்கரையை அளவிட்டார்கள்?  Ask him/her to remember and select the response  அவரை யோசித்துப்ப் பார்த்து பதிலைத் தேர்ந்தெடுக்கச் சொல்லவும் | | | | Within past 6 months ……...1  கடந்த 6 மாதங்களுக்குள்  Within past 6-12 months.….2  கடந்த 6-12 மாதங்களுக்குள்  Within past 1-2years……….3  கடந்த 6-12 வருடங்களுக்குள்  Not within past 2 years……4  கடந்த 2 வருடங்களுக்குள் இல்லை | | | |  | | --- | | |  | | | | | | |
| H12a | If measured in last 12 months where was it done?  கடந்த 12 மாதங்களில் அளவிடப்பட்டது என்றால் எங்கு அளவிடப்பட்டது? | | | | Private doctor’s clinic….1  தனியார் கிளினிக்  Primary Health center / Community Health Center……2  ஆரம்ப அல்லது சமுதாய சுகாதார மையம்  Public/Governement (district/taluk) hospital…3  தாலுகா/மாவட்ட மருத்துவமனை  Private hospital….4  தனியார் மருத்துவமனை  Govt. Medical college/state referral Hospital….5  அரசு மருத்துவ கல்லூரி, மருத்துவமனை  Government AYUSH clinic……6  அரசு ஆயுஷ் கிளினிக்  Private AYUSH clinic….10  தனியார் ஆயுஷ் கிளினிக்  Traditional healer….11  பாரம்பரிய வைத்தியர்  RMP….12  பதிவு செய்யப்பட்ட மருத்துவ பயிற்சியாளர்  Pharmacy….13  மருந்தகம்  Sub centre….14  துணை சுகாதார மையம்  Government Screening camp….15  அரசு பரிசோதனை முகாம்  Private / NGO Screening camp….16  தனியார் / தன்னார்வ தொண்டு நிறுவனம் பரிசோதனை முகாம்  None of the facility visited….17  மேற்கண்ட எதுவும் இல்லை  Don’t know….77  தெரியாது  Refused/ No response ….88  பதிலளிக்க விருப்பமில்லை | | | |  | | --- | | |  | | | | | | |
| H13 | Have you ever been told by a doctor or health worker that you have diabetes?  மருத்துவர் அல்லது மருத்துவ ஊழியர் எப்போதாவது உங்களுக்கு சர்க்கரை வியாதி இருப்பதாக சொல்லியிருக்கிறார்களா ?  Select the response  பதிலைத் தேர்ந்தெடுக்கவும் | | | | Yes…1  ஆம்  No….2  இல்லை | | | |  | | --- | | | *If No, go to H21 இல்லை என்றால். H21க்கு செல்லவும்* | | | | | | |
| H14 | During the past 12 months, have you visited a doctor or other health worker for diabetes?  நீங்கள் கடந்த 12 மாதங்களில் சர்க்கரை வியாதிக்காக மருத்துவர் அல்லது மருத்துவ ஊழியரை பார்த்தீர்களா?  Only for those that have previously been diagnosed withdiabetes  முன்னதாக சர்க்கரை வியாதி இருப்பதாக கண்டறியப்பட்டவர்களுக்கு மட்டும் | | | | Yes…1  ஆம்  No….2  இல்லை | | | |  | | --- | | | *If No, go to H21*  *இல்லை என்றால்.H21க்கு செல்லவும்* | | | | | | |
| H14a | In the past 12 months, which health facility/hospital did you visit most frequently for diabetes?  நீங்கள் கடந்த 12 மாதத்தில் சர்க்கரை வியாதிக்காக பெரும்பாலும் எந்த மருத்துவ வசதி அல்லது மருத்துவமனையை அணுகினீர்கள்? | | | | | Private doctor’s clinic….1  தனியார் கிளினிக்  Primary Health center / Community Health Center……2  ஆரம்ப அல்லது சமுதாய சுகாதார மையம்  Public/Governement (district/taluk) hospital…3  தாலுகா/மாவட்ட மருத்துவமனை  Private hospital….4  தனியார் மருத்துவமனை  Govt. Medical college/state referral Hospital….5  அரசு மருத்துவ கல்லூரி, மருத்துவமனை  Government AYUSH clinic……6  அரசு ஆயுஷ் கிளினிக்  Private AYUSH clinic….10  தனியார் ஆயுஷ் கிளினிக்  Traditional healer….11  பாரம்பரிய வைத்தியர்  RMP….12  பதிவு செய்யப்பட்ட மருத்துவ பயிற்சியாளர்  Pharmacy….13  மருந்தகம்  Sub centre….14  துணை சுகாதார மையம்  None of the facility visited….15  மேற்கண்ட எதுவும் இல்லை  Don’t know….77  தெரியாது  Refused/ No response ….88  பதிலளிக்க விருப்பமில்லை | | --- | | | | |  | | --- | | | If 2,3,5,14 go to H15 | | | | | | |
| H15 | If taken treatment in Government facility such as PHC/CHC/Sub-centre/District/sub district hospital/Govt. medical colleges/state referral hospitals, were the tablets available in the facility itself?  நீங்கள் அரசு மருத்துவ நிலையங்களில் சிகிச்சை பெற்றிருந்ததால் பரிந்துரைக்கப்பட்ட அனைத்து மருந்துகளும் அங்கேயே கிடைத்தனவா?  (ஆரம்ப அல்லது சமுதாய சுகாதார மையம், துணை மத்திய, மாவட்ட மருத்துவமனை அரசு மருத்துவ கல்லூரி, மருத்துவமனை) | | | | Always…..1  எப்பொழுதும்  Sometimes….2  சில நேரங்களில்  Never….3  எப்பொழுதும் இல்லை  Don’t know….8  தெரியாது | | | |  |  | | --- | --- | | | | |  | | | | |
| H16 | On your most recent visit for diabetes, what health facility did you visit?  அண்மையில் சர்க்கரை நோய்க்காக எந்த மருத்துவ வசதியை நீங்கள் அணுகினீர்கள் ? | | | | Private doctor’s clinic….1  தனியார் கிளினிக்  Primary Health center / Community Health Center……2  ஆரம்ப அல்லது சமுதாய சுகாதார மையம்  Public/Governement (district/taluk) hospital…3  தாலுகா/மாவட்ட மருத்துவமனை  Private hospital….4  தனியார் மருத்துவமனை  Govt. Medical college/state referral Hospital….5  அரசு மருத்துவ கல்லூரி, மருத்துவமனை  Government AYUSH clinic……6  அரசு ஆயுஷ் கிளினிக்  Private AYUSH clinic….10  தனியார் ஆயுஷ் கிளினிக்  Traditional healer….11  பாரம்பரிய வைத்தியர்  RMP….12  பதிவு செய்யப்பட்ட மருத்துவ பயிற்சியாளர்  Pharmacy….13  மருந்தகம்  Sub centre….14  துணை சுகாதார மையம்  None of the facility visited….15  மேற்கண்ட எதுவும் இல்லை  Don’t know….77  தெரியாது  Refused/ No response ….88  பதிலளிக்க விருப்பமில்லை | | | |  |  | | --- | --- | | | | |  | | | | |
| H17 | For how long were the drugs (medication) issued for diabetes during the most recent visit?  Drug availability  அண்மையில் உங்கள் சர்க்கரை வியாதிக்காக எத்தனை நாட்களுக்கு மருந்துகளை பெறுகிறீர்கள்? | | | | Less than one week….1  ஒரு வாரத்திற்கும் குறைவான  1 week....2  1 வாரம்  2 weeks….3  2 வாரங்கள்  3 weeks….4  3 வாரங்கள்  1 month….5  1 மாதம் | | | |  | | --- | | | | |  | | | | |
|  | | | |  | | | | |
| H18 | Did you have to pay for any drugs (medication) for diabetes that you received or was prescribed during your most recent visit  அண்மையில், உங்கள் சர்க்கரை வியாதிக்காக மருத்துவர் பரிந்துரைத்த மருந்துகளை வெளியில் பணம் கொடுத்து வாங்கினீர்களா? | | | | Yes…1  ஆம்  No …2  இல்லை | | | |  | | --- | | | |  | | | | | |
| H19 | In the past two weeks, have you taken any drugs (medication) for diabetes prescribed by a doctor or other health worker?  கடந்த 2 வாரங்களில் உங்கள் சர்க்கரை வியாதிக்காக மருத்துவர் அல்லது மருத்துவ ஊழியரால் பரிந்துரைக்கப்பட்ட மருந்துகளை உட்கொண்டீர்களா? | | | | Yes…1  ஆம்  No …2  இல்லை | | | |  | | --- | | | |  | | | | | |
| H20 | What is the reason for not taking medication for diabetes prescribed by a doctor or other health worker?  எந்த காரணத்திற்காக நீங்கள் உங்கள் சர்க்கரை வியாதிக்காக மருத்துவர் அல்லது மருத்துவ ஊழியரால் பரிந்துரைத்த மருந்துகளை எடுத்துக் கொள்ளவில்லை?  (Multiple options possible)  (ஒன்றுக்கு மேற்பட்ட பதில்கள் சாத்தியம்) | | | | | Not prescribed…….1  பரிந்துரைக்கப்படவில்லை  Too expensive/Could not afford….2  மிகவும் விலையுயர்ந்த/வாங்க முடியவில்லை  I didn’t feel sick….3  உடல் நலம் நன்றாக உள்ளது  Side effects….4  பக்க விளைவுகள்  Drugs weren’t working….5  மருந்துகள் வேலை செய்யவில்லை  No health provider /pharmacy nearby….6  மருத்துவர் அல்லது மருத்துவ ஊழியர் / மருந்தகம் அருகில் இல்லை  No one to help me to reach health facility…10  மருத்துவ வசதியை அடைய எனக்கு உதவ யாரும் இல்லை  Too long a wait in health facility….11  மருத்துவமனையில் நீண்ட நேரம் காத்திருத்தல்  Too sick to make the trip….12  உடல்நலக் குறைவால் மருத்துவமனைக்கு செல்ல முடியவில்லை  Do not trust medical care….13  மருத்துவ சேவையை நம்பவில்லை  Do not know where to go….14  எங்கு செல்ல வேண்டும் என்று தெரியவில்லை  Others (Specify) ….15  மற்றவை (குறிப்பிடுக)  Refused/ No response ….88  பதிலளிக்க விருப்பமில்லை | | |  | | --- | | | |  | | | | | |
| **History of raised total cholesterol**  **அதிக கொழுப்பு குறித்த வரலாறு** | | | | | | | | | | | | | | | | |
| H21 | Have you had your cholesterol (fat levels in your blood) measured by a doctor or other health worker?  மருத்துவர் அல்லது மருத்துவ ஊழியர் உங்கள் கொழுப்பு (இரத்தத்தில் உள்ள கொழுப்பு அளவுகள்) அளவுகளை அளந்திருக்கிறார்களா?  Ask the participant to only consider measurements done by a doctor or other health worker  பங்கேற்பாளரை ஒரு மருத்துவர் அல்லது மற்ற மருத்துவ ஊழியர் செய்திருக்கும் அளவீடுகளை மட்டும் கருதச் சொல்லவும் | | Yes…1  ஆம்  No.…2  இல்லை | | | | | |  | | --- | | | *If No, go to*  *H25*  *இல்லை என்றால், H16க்கு செல்லவும்* | | | | | | |
| H22a | Have you ever been told by a doctor or other health worker that you have raised cholesterol? மருத்துவர் அல்லது மருத்துவ ஊழியர் உங்களுக்கு இரத்தத்தில் அதிக அளவு கொழுப்பு இருப்பதாக எப்போதாவது சொல்லியிருக்கிறார்களா? | | Yes…1  ஆம்  No.…2  இல்லை | | | | | |  | | --- | | | *If No, go to H25*  *இல்லை என்றால், H25 க்கு செல்லவும்* | | | | | | |
| H22b | Have you been told by a doctor or other health workerthat you have raised cholesrterol in the past 12 months?  கடந்த 12 மாதங்களில் உங்களுக்கு இரத்தத்தில் அதிக அளவு கொழுப்பு இருப்பதாக சொல்லப்பட்டிருக்கிறதா? | | Yes…1  ஆம்  No.…2  இல்லை | | | | | |  | | --- | | |  | | | | | | |
| H23 | In the past 2 weeks on how many days did you take any oral drugs (medication) for raised cholesterol prescribed by a doctor or other health worker?  கடந்த 2 வாரங்களில், இரத்தத்தில் அதிகமான கொழுப்பிற்க்காக ஒரு மருத்துவர் அல்லது மற்ற மருத்துவ ஊழியர் பரிந்துரைத்த மருந்துகளை நீங்கள் எத்தனை நாட்கள் எடுத்துக் கொண்டிர்கள்?  Ask the participant to only consider drugs for cholesterol prescribed by a doctor or other health worker  பங்கேற்பாளரை மருத்துவர் அல்லது மருத்துவ ஊழியர் கொழுப்பு குறைவதற்காக பரிந்தரைத்த மருந்துகளை மட்டும் கருதச் சொல்லவும் | | Number of days  நாட்களின் எண்ணிக்கை | | | | | |  |  | | --- | --- | | | | |  | | | | |
| **History of Cardiovascular Diseases Cerebro-Vascular Accident**  **இருதய நோய்கள் , மூளை - சார்ந்த** பாதிப்புகள் **குறித்த வரலாறு** | | | | | | | | | | | | | | | | |
| H25 | Have you ever had chest pain (heart related) or a heart attack (angina) or a stroke (cerebrovascular accident or incident) that was diagnosed in a hospital?  உங்களுக்கு நெஞ்சுவலி அல்லது மாரடைப்பு அல்லது பக்கவாதம் இருப்பதாக மருத்துவமனையில் பரிசோதித்து கூறப்பட்டதா? | | | Yes …1  ஆம  No ….2  இல்லை  Don’t Know….7  தெரியாது  No response/refuse….8 | | | | |  | | --- | | | *If No or don’t know go to H28*  இல்லை தெரியாது என்றால் H28  செல்லவும் | | | | | | |
| H25a | If yes at what type of health facility was it diagnosed  ஆம் எனில், எந்த மருத்துவ வசதியில் கண்டறியப்பட்டது? | | | Govt. health facility  அரசு மருத்துவமனை…1  Pvt/NGO health facility  தனியார் / என்ஜிஒ மருத்துவமனை....2 | | | | |  | | --- | | |  | | | | | | |
| H26 | In the last 2 weeks on how many days did you take aspirin to prevent or treat heart disease or stroke? கடந்த 2 வாரத்தில் எத்தனை நாட்கள் இருதய நோய் அல்லது பக்கவாதத்தை தடுப்பதற்கு அல்லது சிகிச்சைக்காக நீங்கள் ஆஸ்பிரின் எடுத்துக் கொள்கிறீர்கள்?  (Check prescription/ tablets)  (மருந்துச்சீட்டு/ மாத்திரைகளை சரிபார்க்கவும்)  For those who were diagnosed to have cardiovascular or Cerebrovascular accident  இருதய நோய் அல்லது ஸ்ட்ரோக் போன்ற மூளை பாதிப்புகள் இருப்பதாக கண்டறியப்பட்டவர்களுக்கு | | | Number of days  நாட்களின் எண்ணிக்கை | | | | |  |  | | --- | --- | | |  | | | | | | |
| H27 | Are you currently taking statins (Lovastatin /Simvastatin/Atorvastatin or any other statin) or any other cholesterol lowering drugs regularly to prevent or treat heart disease or stroke?  நீஙகள் தற்போது இருதய நோய் அல்லது பக்கவாதம் வராமல் தடுப்பதற்கு அல்லது சிகிச்சைக்காக அளிப்பதற்கு நீங்கள் வழக்கமாக ஸ்டாடின்களை (லோவஸ்டாடின் / சிம்வாஸ்டாடின் / அடோவாஸ்டாடின் அல்லது வேறு ஏதாவது ஸ்டாடின்) எடுத்துக் கொள்கிறீர்களா ?  (Check prescription/ tablets)  (மருந்துச்சீட்டு/ மாத்திரைகளை சரிபார்க்கவும்)  “Regularly” means on a daily or almost daily basis  ”வழக்கமானது” என்பது தினசரி அல்லது ஏறக்குறைய தினசரி அடிப்படையில் என்பதைக் குறிக்கிறது | | | Yes…1  ஆம்  No …2  இல்லை | | | | |  | | --- | | |  | | | | | | |

| **Lifestyle Advice வாழ்க்கை முறை குறித்த ஆலோசனை** | | | | |
| --- | --- | --- | --- | --- |
| H28 | During the past one year, has a doctor or other health worker advised any of the following? Select the appropriate response.  Ask the participant to only consider advice from a doctor or other health worker.  கடந்த ஒரு வருடத்தில், மருத்துவர் அல்லது மருத்துவ ஊழியர் பின்வரும் எதாவது அறிவுரை அளித்திருக்கிறாரா?  ஒவ்வொன்றுக்கும் பதிவு செய்யவும்  Select the appropriate response. Ask the participant to only consider advice from a doctor or other health worker  பொருத்தமான பதிலைத் தேர்ந்தெடுக்கவும். ஒரு மருத்துவரிடமிருந்து அல்லது மற்ற மருத்துவ ஊழியரிடமிருந்து பெறப்பட்ட அறிவுரையை மட்டும் கருதவும்; | | |  |
| H28a | Against starting smoking tobacco or quitting smoking  புகையிலை புகைக்கத் தொடங்குவதற்கு எதிராக அல்லது புகைப்பதை கைவிடுவது | Yes…1  ஆம்  No.…2  இல்லை  மருத்துவர் அல்லது மற்ற மருத்துவ ஊழியரை சந்திக்கவில்லை…9 | |  | | --- | | If did not meet any health worker or doctor, GO TO CX1 மருத்துவர் அல்லது மற்ற மருத்துவ ஊழியரை சந்திக்கவில்லை என்றால், CX1 க்கு செல்லவும் |
| H28b | Against start using smokeless tobacco or quitting smokeless tobacco  புகையில்லாத புகையிலையை உபயோகிக்கத் தொடங்குவதற்கு எதிராக அல்லது புகையில்லாத புகையிலையை கைவிடுவது | Yes…1  ஆம்  No…2  இல்லை | |  | | --- | |  |
| H28c | Against start taking alcohol or decrease intake or alcohol cessation  மதுபானம் குடிக்கத் தொடங்குவதற்கு எதிராக அல்லது குடிப்பதை குறைப்பது அல்லது மதுபானத்தை நிறுத்துவது | Yes…1  ஆம்  No.…2  இல்லை | |  | | --- | |  |
| H28d | Reduce salt in your diet  உங்கள் உணவில் உப்பைக் குறைப்பது | Yes…1  ஆம்  No …2  இல்லை | |  | | --- | |  |
| H28e | Increase fruit and vegetable intake in your diet  உங்கள் உணவில் பழம் மற்றும் காய்கறிகள் எடுத்துக் கொள்வதை அதிகரிப்பது | Yes…1  ஆம்  No …2  இல்லை | |  | | --- | |  |
| H28f | Reduce fat in your diet  உங்கள் உணவில் கொழுப்பைக் குறைப்பது | Yes…1  ஆம்  No …2  இல்லை | |  | | --- | |  |
| H28g | Increase physical activity  உடல் செயல்பாடுகளை அதிகரிப்பது குறித்து | Yes…1  ஆம்  No …2  இல்லை | |  | | --- | |  |
| H28h | Maintain a healthy body weight or lose weight  ஆரோக்கியமான உடல் எடையைப் பராமரிப்பது அல்லது எடையை இழப்பது | Yes…1  ஆம்  No …2  இல்லை | |  | | --- | |  |
| H28i | Practice Yoga  யோகா பயிற்சி | Yes…1  ஆம்  No …2  இல்லை | |  | | --- | |  |

| **Cancer Screening (To be administered to those above 30 years of age only)**  **புற்றுநோய்க்கான ஆரம்ப கட்ட பரிசோதனை (30 வயதிற்கு மேற்பட்டவர்களிடம் மட்டுமே கேட்கப்பட வேண்டும்)** | | | | | | | |
| --- | --- | --- | --- | --- | --- | --- | --- |
| Certain examination or tests are done to look for cancer in its early stage and this is known as cancer screening. Different cancers have different screening methods. You may have been subjected to any of the methods. So please try to recall if any of the methods I shall be describing below in the relevant section were done for you.  புற்றுநோயை அதன் ஆரம்ப நிலையில் கண்டறிவதற்கு சில ஆய்வுகள் மற்றும் பரிசோதனைகள் செய்யப்படுகின்றன. இவை புற்றுநோய் கண்டறிவதற்கான ஆய்வு பரிசோதனைகள் ஆகும். புற்றுநோய்களுக்கான ஆய்வு பரிசோதனைகள் ஒவ்வொரு புற்றுநோய்க்கும் வேறுபடும். பின்வரும் பரிசோதனைகளில் நீங்கள் ஏதேனும் ஒரு பரிசோதனைக்கு உட்படுதபட்டிருக்கலாம். அதனால் நீங்கள் கூறும் பின்வரும் பரிசோதனை முறைகளில் ஏதேனும் ஒன்று உங்களுக்கு செய்யபட்டிருக்கிறதா என நினைவு கூறவும்.  Read this opening statement out loud. It should not be omitted.  இந்த தொடக்க வாக்கியத்தை சப்தமாக வாசிக்கவும். இதனை விட்டு விடக்கூடாது பதிலளிப்பவர் ஆண் எனில் பகுதி 2 செல்லவும் | | | | | | | |
|  | **Oral cancer screening** வாய் புற்றுநோய் கண்டறிதல் | | | | | | |
| CX1 | Have you ever had any doctor or dentist examined your oral cavity to look for **early signs of cancer**?  வாய் புற்றுநோயின் ஆரம்ப கட்ட அறிகுறிகள் இருக்கிறதா என்பதைப் அறிவதற்கு பொது /பல் மருத்துவர் அல்லது மருத்துவ ஊழியர் பரிசோதித்திருக்கிறார்களா?  Remember that it does not include oral examination for a dental problem or other non-cancer related problems.  இதில் பல் வியாதிக்கான வாய் பரிசோதனை அல்லது மற்ற புற்று-நோயுடன் தொடர்பான பிரச்சனைகள் உள்ளடங்காது என்பதை நினைவில் வைத்துக் கொள்ளவும். | Yes …1  ஆம்  No ….2  இல்லை  Don’t Know….7  தெரியாது  No response/ Refused….8  பதில் இல்லை/ மறுத்தல் | | | |  |  | | --- | --- | | | *If respondent is a Male go to Section 2 M0*  பதிலளிப்பவர் ஆண் எனில் பகுதி 2 M0 செல்லவும் |
|  | **Breast Cancer screening** (For Women only)  **மார்பக புற்றுநோய்க்கான ஆரம்ப கட்ட ப**ரி**சோதனை (பெண்களுக்கு மட்டும்)** | | | | | | |
| CX2 | Have you ever performed Breast Self-Examination to look for breast cancer?  மார்பக புற்றுநோய் இருக்கிறதா என்பதைக் கண்டறிவதற்காக நீங்கள் எப்போதாவது மார்பக சுய-பரிசோதனை செய்திருக்கிறீர்களா? | | | Yes…1  ஆம்  No …2 இல்லை  Don’t Know….7  தெரியாது  No response/ Refused….8  பதில் இல்லை/ மறுத்தல் | | |  | | --- | |  |
| CX3 | Have you ever had any breast examination by a doctor to look for Breast Cancer?  மார்பக புற்றுநோயைப் இருக்கிறதா எனப் பார்ப்பதற்கு நீங்கள் எப்போதாவது மருத்துவரிடம் மார்பக பரிசோதனை செய்து கொண்டிருக்கிறீர்களா? | | | Yes…1  ஆம்  No.…2 இல்லை  Don’t Know….7  தெரியாது  No response/ Refused….8  பதில் இல்லை/ மறுத்தல் | | |  | | --- | |  |
| CX4 | Have you ever undergone an ultrasound of breast or mammography?  நீங்கள் எப்போதாவது மார்பக அல்ட்ராசவுன்டு அல்லது மேமோக்ராஃபி செய்திருக்கிறீர்களா? | | | Yes…1  ஆம்  No …2 இல்லை  Don’t Know….7  தெரியாது  No response/ Refused….8  பதில் இல்லை/ மறுத்தல் | | |  | | --- | |  |
|  | Cervical Cancer Screening (For Women only)  **கர்ப்பப்பைவாய் புற்றுநோய்க்கான ஆரம்பக்கட்ட பரிசோதனை (பெண்களுக்கு மட்டும்)** | | | | | | |
| Screening tests for cervical cancer prevention can be done in different ways like Visual Inspection with Acetic Acid/vinegar (VIA), pap smear & Human Papillomavirus (HPV) test.  **கர்ப்பப்பைவாய்** புற்றுநோயைத் தடுப்பதற்கான ஆரம்ப கட்ட பரிசோதனைகள் வெவ்வேறு முறைகளில் செய்யப்படுகிறது. அவை அசிடிக் ஆசிட் (வினிகர்) உபயோகித்து கண்டறிவது, பேப் ஸ்மியர் பரிசோதனை மற்றும் ஹியூமன் பாபிலேமாவைரஸ் (ஹெச் பி வி) பரிசோதனை போன்றவைகள் ஆகும்.   - VIA is an inspection of the surface of the uterine cervix after acetic acid (or vinegar) has been applied to it   வி ஐ ஏ என்பது அசிடிக் ஆசிட் (வினிகர்) பயன்படுத்தி கருப்பை வாயின் மேற்புரத்தைப் பரிசோதிப்பதாகும்   - For both pap smear & HPV test, a doctor / nurse uses a swab to wipe from inside your vagina, take a sample and send it to a laboratory   பேப் ஸ்மியர் மற்றும் ஹெச் பி வி பரிசோதனைக்காக, உங்கள் பெண் உறுப்பு பகுதியின் உள்ளிலிருந்து, ஒரு ஸ்வாபை உபயோகித்து ஒரு மருத்துவர் / நர்ஸ் ஒரு மாதிரியை (sample) எடுத்து அதனை ஆய்வுகூடத்திற்கு பரிசோதனைக்கு அனுப்பி இருப்பார்கள்  Read this opening statement out loud. It should not be omitted  இந்த தொடக்க வாக்கியத்தை சப்தமாக வாசிக்கவும். இதனை விட்டு விடக் கூடாது. | | | | | | | |
| CX5 | Have you ever had a screening test for cervical cancer, using any of these methods described above?  மேலே குறிப்பிடப்பட்ட முறைகள் எதையாவது உபயோகித்து நீங்கள் கர்ப்பப்பைவாய் புற்றுநோய்கான ஒரு ஆரம்ப கட்ட பரிசோதனையை எப்போதாவது செய்திருக்கிறீர்களா?  Remember that it includes both self-sought as well as opportunistic screening for cervical cancer.  சுயமாக செய்து கொண்ட மற்றும் மருத்தவமனைக்கு வேறு காரணத்திற்க்காக சென்ற பொழுது மருத்துவர் அல்லது மருத்துவ பணியாளரின் அறிவுரையின் பேரில் செய்து கொண்ட இரண்டு விதமான பரிசோதனைகளும் இதில் அடங்கும். | | Yes …1  ஆம  No ….2  இல்லை  Don’t Know….7  தெரியாது  Refused….8  பதில் இல்லை/ மறுத்தல் | | | |  | | --- | |  |

| **Section 2. Physical Measurement: உடல் ரீதியிலான அளவீடு/;** | | | | | | | |  |
| --- | --- | --- | --- | --- | --- | --- | --- | --- |
| M0 | | | Are you willing to undergo anthropometric measurements?  நீங்கள் உடல்ரீதியான அளவீடுகளை செய்து கொள்வதற்குத் தயாராக இருக்கிறீர்களா? | | Yes.…1  ஆம்  No…2  இல்லை | |  | If no go to  BC1  இல்லை எனில்எனில் க்குசெல்லவும் |
|  | | | | | | | | |
| B.P. Reading 2  இரத்த அழுத்த அளவீடு 2  *Enter 888 in all field if participant has refused* | | | | | | | |  |
| M2a | Systolic blood pressure (mmHg)  சிஸ்டாலிக் இரத்த அழுத்தம் | | | Systolic (mmHg)  சிஸ்டாலிக் | | |  |  |  | | --- | --- | --- | | |  |
| M2b | Diastolic blood pressure (mmHg)  டையாஸ்டோலிக் இரத்த அழுத்தம் | | | Diastolic (mmHg)  டையாஸ்டோலிக் | | |  |  |  | | --- | --- | --- | | |  |
| M2c | Pulse Rate Reading 2  இதயத்துடிப்பு அளவீடு 2  Record the reading  அளவீட்டைப் பதிவு செய்யவும் | | | Beats/min  நாடிதுடிப்புகள் / நிமிடத்திற்கு | | |  |  |  | | --- | --- | --- | | |  |
| B.P. Reading 3  இரத்த அழுத்த அளவீடு *3*  *Enter 888 in all field if participant has refused* | | | | | | | |  |
| M3a | Systolic blood pressure (mmHg)  சிஸ்டாலிக் இரத்த அழுத்தம் | | | Systolic (mmHg)  சிஸ்டாலிக் | | |  |  |  | | --- | --- | --- | | |  |
| M3b | Diastolic blood pressure (mmHg)  டையாஸ்டோலிக் இரத்த அழுத்தம் | | | Diastolic (mmHg)  டையாஸ்டோலிக் | | |  |  |  | | --- | --- | --- | | |  |
| M3c | Pulse Rate Reading 3  இதயத்துடிப்பு அளவீடு 3  Record the reading  அளவீட்டைப் பதிவு செய்யவும் | | | Beats/min  நாடிதுடிப்புகள் / நிமிடத்திற்கு | | |  |  |  | | --- | --- | --- | | |  |
| **Height and Weight and Waist circumference** உயரம் எடை மற்றும் இடுப்பின் சுற்றளவு | | | | | | | |  |
| M4 | | Height  உயரம்    Record participant's height in cm with one decimal point  Enter '888' if participant has refused.  ஒரு தசம புள்ளியுடன் செ.மீல் பங்கேற்பாளரின் உயரத்தைப் பதிவு செய்யவும். | | In Centimeter(cm)…  சென்டிமீட்டரில் (செ.மீ) | | |  |  |  | **.** |  | | --- | --- | --- | --- | --- | | |  |
| M5 | | Weight  எடை  Record participant's weight in kg with one decimal point.  Enter '888' if participant has refused.  ஒரு தசம புள்ளியுடன் செ.மீ ல் பங்கேற்பாளரின் எடையைப் பதிவு செய்யவும். | | In Kilograms (kg)…  கிலோகிராம்களில்  (கிலோ) | | |  |  |  | **.** |  | | --- | --- | --- | --- | --- | | |  |
| M6 | | Waist circumference  இடை சுற்றளவு  Record participant's waist circumference in centimetres with one decimal point.  Enter '888' if participant has refused.  ஒரு தசம புள்ளியுடன் செ.மீ ல் பங்கேற்பாளரின் இடைச் சுற்றளவைப் பதிவு செய்யவும். | | In Centimeter(cm)  சென்டிமீட்டரில் (செ.மீ) | | |  |  |  | **.** |  | | --- | --- | --- | --- | --- | | |  |

|  |
| --- |

| **Blood Pressure and Pulse Rate இரத்த அழுத்தம் மற்றும் நாடி துடிப்பு** |
| --- |
| - *For Blood Pressure (BP) measurement the respondent must be comfortable and if the respondent had been exerting then he/she must rest for at least 5 minutes.*   இரத்த அழுத்த அளவீடுகளுக்கு, பங்கேற்பாளர் சௌகரியமாக அமர வேண்டும் மற்றும் பங்கேற்பாளர் ஏதேனும் கடின வேலை செய்து கொண்டிருந்தால் அவர் குறைந்தபட்சம் 5 நிமிடங்களுக்கு ஓய்வெடுக்க வேண்டும்.   - *There must be at least 5 minutes gap between two consecutive readings.*   *இரண்டு தொடர்ச்சியான அளவீடுகள் இடையே குறைந்தபட்சம் 5 நிமிடங்கள் இடைவெளி இருக்க* வேண்டும்.  *If the respondent refuses any of the measurement write 888*  பங்கேற்பாளர் *ஏதாவது அளவீடுகளைச் செய்வதற்கு மறுத்தால் 888 என எழுதவும்* |

| **Section 3. Biochemical Measurement** உயிர் வேதியியல் அளவீடு | | | | |
| --- | --- | --- | --- | --- |
| BC1 | Did participant provide blood for blood sugar measurement பங்கேற்பாளர் பங்கேற்பாளர் இரத்தத்தில் உள்ள சர்க்கரை அளவை அறிய இரத்த மாதிரி கொடுத்துள்ளாரா? | Yes….1  ஆம்  No…2 இல்லை | |  | | --- | | *If No, go to Z1* |
| BC2 | During the past 8-12 hours have you had anything to eat or drink, other than water?  கடந்த 8-12 மணி நேரத்தில் தண்ணீரைத் தவிர ஏதாவது குடித்தீர்களா அல்லது சாப்பிட்டீர்களா? | Yes.…1  ஆம்  No.…2 இல்லை | |  | | --- | |  |
| BC3 | Date of last meal/ drink  கடைசியாக உட்கொண்ட நாள் |  | |  |  |  |  |  |  | | --- | --- | --- | --- | --- | --- | |  |
| BC4 | Time of last meal / drink  கடைசியாக உட்கொண்ட நேரம் |  | |  |  |  |  |  | | --- | --- | --- | --- | --- | |  |
| BC5 | Today, have you taken insulin or other drugs (medication) that have been prescribed by a doctor or other health worker for raised blood glucose?  இன்று சர்க்கரை வியாதிக்காக மருத்துவர் அல்லது மருத்துவ ஊழியர் பரிந்துரைத்த இன்சுலின் அல்லது மற்ற மருந்துகளை எடுத்தீர்களா? | Yes ...…1  ஆம்  No…2 இல்லை  Not applicable….9  பொருந்தாது | |  | | --- | |  |
| BC6 | Fasting Blood Glucose level(mg/dl)  காலை உணவிற்கு முன் எடுக்கப்படும் இரத்த சர்க்கரை அளவு (mg/dl) |  | |  |  |  | | --- | --- | --- | |  |

|  | **Location** |  |
| --- | --- | --- |
| Z1 | Record GPS coordinates |  |
|  | No GPS coordinates entered. Please try again. |  |
| Z2 | Number of visits made | One……………….1  Two……………….2  Three…………….3 |
| Z3 | Outcome | | Refused not filled ………….1 | | --- | | Locked not filled……………2 | | Form incomplete due to midway refusal...3 | | Form incomplete as person had to leave midway………………….4 | | Form complete….5 | | Form incomplete need to revisit….6 | |
