# Supplementary material for: Prevalence of Noncommunicable Disease (NCDs) risk factors in Tamil Nadu: Tamil Nadu STEPS Survey (TN STEPS), 2020
Source: PLoS One. 2024 May 8;19(5):e0298340. doi: 10.1371/journal.pone.0298340 (PMC11078398; doi:10.1371/journal.pone.0298340)
Supplement: Attachment — Submitted filename: review of PONE-D-23-15618.pdf [file pone.0298340.s006.pdf]

Dear Editor,

Thank you for the opportunity to review the manuscript entitled “Prevalence of Noncommunicable Disease (NCDs) Risk Factors in Tamil Nadu: Tamil Nadu STEPS Survey (TN STEPS), 2020.”. I read the manuscript with great interest and found it highly relevant. However, I have the following suggestions for further improvement.

Abstract:

1. Study aim(s) is/are missing in the abstract.
2. Methods line 14: The authors mentioned calculating adjusted prevalence ratios of diabetes and hypertension. Reading the introduction, I had an impression that several NCDs risk factors will be assessed. The results also provide prevalence estimates for several factors. So the statistical analysis under the method in the abstract needs revisiting and being comprehensive to include other factors as well.

Introduction

3. Literature review could be improved by providing information on the prevalence of NCDs risk factors at the local level. I understand that no previous state-wide studies have been conducted, but I wonder if there are small-scale local studies that could provide some estimates of NCDs risk factor prevalence in Tamil Nadu.
4. A brief statement on why existing nationwide data is insufficient may provide context for readers to understand inter-state diversity in India. In other words, why is it important to get estimates at the state level when national-level data exists? The authors should understand that readers may not be familiar with the heterogeneity (i.e., socio-economy, culture, and development) between states in India that could potentially impact the prevalence of diseases and health outcomes.
5. Why were predictors of only two factors, i.e., diabetes and hypertension, determined? I believe the authors have sound justification for doing that, but that justification is not articulated for readers.

Methods

6. Study Setting and Population: Why those 70 and above were excluded from participation? Please provide your justification.
7. Sampling strategy: International readers may not be familiar with concepts like ward, so I suggest adding a brief context for comprehension. Likewise, KISH method is not self-explanatory, so a brief explanation will help the reader to understand how sampling was done.
8. Data collection procedure: please add what the two-week training for data collection teams included.
9. Lines 85-87 are confusing and contradictory. The authors mentioned, “The team members enumerated all the eligible individuals in the selected households.” The following line then indicated, “Using the KISH technique, the app had an in-built feature to select one participant

per household automatically." I am confused if data was collected from everybody or a single person in the household.

10. Data analysis: For the regression models, was multicollinearity tested and how the model fit was determined?
11. The operational definition of variables in Table 1 is very helpful, but it would be helpful to know what was asked to participants and/or how each was measured. The method section should have a section on variables measurements, but if that's not feasible, it should be in the supplemental text or table.

Discussion

12. The discussion section lacks possible explanations for observed findings. For example, what explains the high burden of NCDs risk factors overall, and what mechanism supports differences between males and females?

Minor comment:

13. The manuscript needs proofreading for grammar issues. For example: in several places, there are inappropriate cases. Abbreviation (e.g., Govt of TN) is used without specifying at first use.
