# Supplementary material for: Prevalence of Noncommunicable Disease (NCDs) risk factors in Tamil Nadu: Tamil Nadu STEPS Survey (TN STEPS), 2020
Source: PLoS One. 2024 May 8;19(5):e0298340. doi: 10.1371/journal.pone.0298340 (PMC11078398; doi:10.1371/journal.pone.0298340)
Supplement: Attachment — Submitted filename: Responses to the comments.docx [file pone.0298340.s007.docx]

**Responses to the comments:**

**Additional Editor Comments:**

| S.NO | Comments/Suggestions from the reviewer | Responses | Page number in which changes were done |
| --- | --- | --- | --- |
| 1 | In the abstract, rather than a comparable way of presenting the conclusions, it would be ideal to showcase what are the major ones and how big it is in your state and what factors are primarily associated with, and drawing the conclusion in that line would be more appropriate. The conclusion in the main body of the manuscript seems more logical, try to align the conclusion of abstract as well to that. | We thank the editor for the comment. We have incorporated your suggestions into the revised version. Our conclusion in the abstract has been modified to match our conclusion in the body of the paper. | Page 2, lines 56-60 |
| 2 | Methods: What does it mean by abruptly stopped the survey, at what stage it was stopped and what happened after that? | We had to stop the survey on 21st March 2020, due to the national lockdown imposed as a response to the COVID-19 pandemic. We had covered 82% of the sample size by the time of the lockdown. Due to the pandemic, even after the lockdown restrictions were removed, the technical advisory committee for the survey at the state advised not to resume data collection. | page 13, line-229-31 |
| 3 | Sample size. There is a calculation fallacy in the sample size calculation. The initial sample size of 578 is fine with the parameters and the value used. Then in the next step it has to be multiplied by 8 (to cover 8 age sex groups as per the description), which means the total sample will be 4624. This also looks fine. Now where is the adjustment for 80% response rate (assumption of 20% non-response). I am sure authors are aware about the response rate adjustment. The required sample size for your survey as per the design is 4624, now you will have to increase the sample size by adjusting the non-response which will lead to 5780. If you calculate the same using Steps Sample size calculator you will get 5762 as final sample size with your description. That minimal difference of 5762 and 5780 doesn’t make a difference, but the missing adjustment of non-response assumption is a complete fallacy. However, when I see the results, you have only used 3 age groups in analysis. Please see this thoroughly and update the manuscript accordingly. There is a serious mismatch here. | We thank the editor for the comment. While calculating sample size we followed the same logic as explained by the editor. We adjusted for a non-response rate of 20% by using the formula Final sample size= effective sample size/ (1-non-response rate anticipated). We arrived at a required sample size of 5780. We decided to cover this sample size by sampling 30 adults from 193 clusters (merged villages and wards) which gives a total of 5790.  We understand that we had not articulated this well in the manuscript and we have rephrased it in the revised manuscript.  Also, though we had calculated sample size for providing precise estimates for 4 age groups, since we could not cover the planned sample size owing to the COVID-19 lockdown, we have given the results only for 2 age groups, after discussing with the stakeholders. | page 6, line-128-133 |

**Reviewer 1:**

| **S.NO** | **Comments/Suggestions from reviewer** | **Responses** | **Page number in which changes was done** |
| --- | --- | --- | --- |
| 1 | Study aim(s) is/are missing in the abstract. | Thank you for the comment, we have incorporated all of your suggestions into my revision. The Aim and methods in the abstract have been changed accordingly. We have provided prevalence estimates of several risk factors, however, predictors of diabetes and hypertension were assessed only for diabetes and hypertension. We acknowledge the importance of providing context and understanding the inter-state diversity in India. We have considered your suggestions and have made modifications | page 2, line-33-35 |
| 2 | Methods line 14: The authors mentioned calculating adjusted prevalence ratios of diabetes and hypertension. Reading the introduction, I had an impression that several NCDs risk factors will be assessed. The results also provide prevalence estimates for several factors. So the statistical analysis under the method in the abstract needs revisiting and being comprehensive to include other factors as well. | We have provided prevalence estimates of several risk factors, however, the predictors of diabetes and hypertension were of particular interest to the stakeholders from the state health department. We have provided predictors for other risk factors in the supplementary materials. | page 22, line-230 to 233 |
| 3 | Literature review could be improved by providing information on the prevalence of NCDs risk factors at the local level. I understand that no previous state-wide studies have been conducted, but I wonder if there are small-scale local studies that could provide some estimates of NCDs risk factor prevalence in Tamil Nadu | We thank the reviewer for the suggestion. We have added the following in the introduction to explain the same. "India is a huge country with diversity in socioeconomic status, cultural practices, and level of epidemiological transition. Though nationwide estimates are available, a state-specific estimate is important for planning as Tamil Nadu is one of the few states with a high level of epidemiological transition in India." | page 4, line-74-77 |
| 4 | A brief statement on why existing nationwide data is insufficient may provide context for readers to understand inter-state diversity in India. In other words, why is it important to get estimates at the state level when national-level data exists? The authors should understand that readers may not be familiar with the heterogeneity (i.e., socioeconomy, culture, and development) between states in India that could potentially impact the prevalence of diseases and health outcomes. | We acknowledge the importance of providing context and understanding the inter-state diversity in India. We have considered your suggestions and have made modifications.  We have explained how epidemiological transition is not uniform in all states and how differences in socio-cultural practices are also key to this. | page 4, line-72-77 |
| 5 | Why were predictors of only two factors, i.e., diabetes and hypertension, determined? I believe the authors have sound justification for doing that, but that justification is not articulated for readers. | We have provided prevalence estimates of several risk factors, however, the predictors of diabetes and hypertension were of particular interest to the stakeholders from the state health department. We have provided predictors for other risk factors in the supplementary materials. | page 22, line-226 to 228 |
| 6 | Methods: Study Setting and Population: Why those 70 and above were excluded from participation? Please provide your justification. | The standard WHO NCD STEPS Survey methods indicate including risk factor surveys for adults aged 18 and 69. Therefore to ensure comparability with other studies we have included the same age group. Also, globally risk factors surveillance for NCD is done to plan for strategies to reduce premature mortality (death less than 70 years of age). Therefore, our study has also focused on the age group 18-69. | page 6, line-115 to 117 |
| 7 | Sampling strategy: International readers may not be familiar with concepts like ward, so I suggest adding a brief context for comprehension. Likewise, KISH method is not self-explanatory, so a brief explanation will help the reader to understand how sampling was done | We acknowledge that the concept of wards and the KISH technique should be explained. We have made the necessary modifications | page 7, line-136 & 143 |
| 8 | Data collection procedure: please add what the two-week training for data collection teams included. | During the first week Q by Q (Question by Question) training was done for the data collectors. Proper ways of measuring study parameters such as anthropometry (height, weight, waist circumference), blood pressure, and capillary blood glucose measurements were taught. The data collectors were trained on how to use the ODK app for mapping of villages and enumeration of primary sampling units, how to enter data, and take consent. During the second week of the training, mock sites were chosen, and a few sample/dummy participants were selected to observe and oversee the field-level work. | page 8, line-174 -181 |
| 9 | Lines 85-87 are confusing and contradictory. The authors mentioned, “The team members enumerated all the eligible individuals in the selected households.” The following line then indicated, “Using the KISH technique, the app had an in-built feature to select one participant per household automatically.” I am confused if data was collected from everybody or a single person in the household. | The data was collected from one individual per selected household. The sampling procedure involves village/wards as the primary sampling unit (PSU), 193 PSUs were selected using probability proportional to size systematic sampling method (PPSS). Next, from each PSU we selected 1 Census Enumeration Block (CEB) was selected using the probability proportional to size (PPS). Household listing was done for the selected CEB. This list was then uploaded to the central server. Using systematic random sampling the ODK app selected 30 households from each CEB. Household enumeration of the 30 households was carried out, and after the enumeration of the eligible adults aged 18-69, one individual from each household will be selected automatically by the ODK app using the KISH method and that individual will be interviewed accordingly. | page 9, line-189 to 192 |
| 10 | Data analysis: For the regression models, was multicollinearity tested and how the model fit was determined? | We thank the reviewer for the question. We have checked for multicollinearity in the model using the Variation Inflation Factor (VIF) for all independent variables. We also determined the fitness of the model using the likelihood ratio test (lrtest) test of the full model and the nested model. We have given details of the VIF for all independent variables included in the model in the supplementary table for reference. | page 12, line-228 to 230 |
| 11 | The operational definition of variables in Table 1 is very helpful, but it would be helpful to know what was asked to participants and/or how each was measured. The method section should have a section on variables measurements, but if that’s not feasible, it should be in the supplemental text or table. Discussion | Thank you for the comment we have added supplementary table of the questionnaire for the study | page 9, line-201 |
| 12 | The discussion section lacks possible explanations for observed findings. For example, what explains the high burden of NCDs risk factors overall, and what mechanism supports differences between males and females? Minor comment: | We thank the reviewer for the comment. We have explained that there has been rapid urbansiation and globalisation that has happened in the state of Tamil Nadu, which explains the epidemiological transition in the state. This is the predominant reason for a high burden of NCDs seen in the state. We have also explained how socio-cultural norms and peer pressure are the reasons for the gender differences seen among men and women, especially for risk factors like smoking and alcohol use. | page 27, line-339 to 346 |
| 13 | The manuscript needs proofreading for grammar issues. For example: in several places, there are inappropriate cases. Abbreviation (e.g., Govt of TN) is used without specifying at first use. | We have proofread for grammatical errors and changed the abbreviation written in the title to Tamil Nadu instead of TN and other abbreviations have also been expanded accordingly. | page 1, line-11,14,16 |

**Reviewer 2:**

| **S.NO** | **Comments/Suggestions from reviewer** | **Responses** | **Page number in which changes were done** |
| --- | --- | --- | --- |
| 1 | Overall, the manuscript abstract effectively presents the background, methods, key findings, and implications of the study. Please add the relevant numbers and statistics (95% confidence intervals etc.) to support your statements in the results section of the abstract | Thank you for the comment. We have incorporated your suggestions into the revised version | page 2, lines 48 to 52 |
| 2 | Methods: A representative cross-sectional study was conducted among 18-69-year-old adults in 10 Tamil Nadu in 2020- representative in terms of national, provincial or what? | Thank you for the comment.  The study sampling methods were designed to ensure that the sample is representative of the state of Tamil Nadu.  The sampling method was multi-stage sampling. The primary sampling units were villages/wards (administrative units in urban areas). The second stage of sampling involved sampling census enumeration blocks (units used for sampling in the National census; each CEB has roughly about 150-200 households). Within each selected block 30 households were selected using Systematic random sampling. In each household, one individual was selected using the KISH method. | page 6, 135 to 149 |
| 3 | Main body: Introduction: Paragraph 1- Please shows the global landscape of NCD highlighting its prevalence, morbidity, and mortality on worldwide scale and narrow your focus to India, exploring the specific challenges and implications faced by the country in relation to prevalence of noncommunicable diseases (NCDs) and associated risk factors. | Thank you for your comment, we have incorporated your suggestions into our revised manuscript by adding the global landscape of NCD. | page 4, line 71 |
| 4 | Results It is important to acknowledge that you covered 82% of the intended sample size of 4624 participants, with a final sample size of 3800 individuals. However, it is crucial to address the potential implications of the reduced sample size on the generalizability and statistical power of your findings. Consider discussing any potential biases that may arise from the incomplete coverage of the intended sample and any steps taken to mitigate such biases. What was the response rate? I couldn’t locate where the actual response rate is presented. Please also clarify how it was calculated. | We thank the reviewer for the comment. Since the study did not cover the intended sample size, the precision of the prevalence estimates will be reduced if analyses were to be done for all the intended age and gender strata (4 age strata and 2 gender: so, a total of 8 strata). This was discussed with the stakeholders, and it was decided that analysis will be done only for two age groups (a total of 4 strata). The sample size that was covered was adequate to precisely estimate the prevalence of NCD risk factors for 4 strata.  The overall response rate was 92%. We have added a table comparing the characteristics of respondents and non-respondents. Though the proportion of men and younger individuals were higher in the non-respondent’s group, it might not affect the external validity to a great extent considering that the overall response rate was 92%. | Change on page 13, lines 238 to 242 |
| 5 | Please add 95% CI before range of result, check result section for e.g line 153 | We have added CI before the range of the result. | page 16 |
| 6 | Discussion The comparisons with studies undertaken in other countries are useful. Please compare your results with those from other studies conducted outside India. It seems that the discussion section is missing the discussion of variables that reached significance in the multivariable analysis for the purpose of comparing them to studies conducted outside India. MPOWER and SAFER strategies what are they? – please check and provide full form of all abbreviated form in initial throughout the manuscript. | We thank the reviewer for the suggestions. We have included information about NCD risk factor prevalence from other Low-and Middle-Income Countries (LMICs) in the discussion section. We have also expanded the section on MPOWER and SAFER strategies. | page 28 line 389, page 29 line 411, |
