# Supplementary material for: Prevalence of Noncommunicable Disease (NCDs) risk factors in Tamil Nadu: Tamil Nadu STEPS Survey (TN STEPS), 2020
Source: PLoS One. 2024 May 8;19(5):e0298340. doi: 10.1371/journal.pone.0298340 (PMC11078398; doi:10.1371/journal.pone.0298340)
Supplement: Attachment — Submitted filename: Response_to_comment_TN_STEP_14jan24.docx [file pone.0298340.s008.docx]

**Responses to the comments:**

**Additional Editor Comments:**

| S.NO | Comments/Suggestions from the reviewer | Responses | Page number in which changes were done |
| --- | --- | --- | --- |
| 1 | A new author has been added to the authors list. Please provide justification/clarification for the same. | The new author has played a critical role in the conceptualization of the study. He has also made vital contributions to preparing the data collection and supervision tools. He reviewed the final manuscript and gave critical inputs. His name was missed due to a clerical error during the initial submission.  Therefore, we have added his name to the revised submission. | Page 1 in the track version |
| 2 | The way CIs are mentioned appears to be done in quite a rush, please check it. I noticed in the abstract but check throughout, it has to be mentioned as 95%CI: 36.4-42.0. You don't need to write % in CI. | We thank the editor for the comment. We have incorporated your suggestions into the revised version. | Page 2,3  And entire Results -Page 13-33 in the track version |
| 3 | Line 97 of track changes version - … had an overall prevalence of 32.44%.... please be consistent in writing digits after decimal. Here you have written 2 digits, but elsewhere single digit. I would suggest using single digit throughout. | We appreciate your valuable feedback.  We acknowledge the inconsistency in the representation of digits after the decimal point. We agree with your suggestion to maintain uniformity and use a single digit throughout the manuscript. | Page 5, line-89. Page 18: Table 3 in the track version. |
| 4 | The first para of the results section where you talk about response rate etc… you have not updated the information as per the comment on sample size. | We thank the editor for the comment. We have carefully revised the paragraph to reflect the appropriate changes, ensuring consistency with the sample size. | Page no 13, Lines 235-243 in the track version |
| 5 | Please review the whole paper thoroughly for minor issues of copy edits, language, spelling and inconsistency. | We thank the editor for the comment. We have revised the manuscript by doing spelling and grammar checks. | Throughout the manuscript |
